# Comparative Study of Spent Sand Reclamation from Binder Jetting and Conventional Foundry Technologies

**DOI:** 10.3390/ma19194219

**Published:** 2026-10-03

**Authors:** Rafał Dańko, Angelika Kmita, Daniel Gruszka

**Affiliations:** 1 Faculty of Foundry Engineering, AGH University of Krakow, Adama Mickiewicza 30, 30-059 Krakow, Poland; dgruszka@agh.edu.pl; 2 Academic Centre for Materials and Nanotechnology, AGH University of Krakow, Adama Mickiewicza 30, 30-059 Krakow, Poland; akmita@agh.edu.pl

**Keywords:** binder jetting, additive manufacturing, spent foundry sand, sand reclamation, mechanical reclamation, thermal reclamation, furfuryl resin

## Abstract

The growing use of binder jetting in foundry practice has increased the need for effective recovery of the fine silica sand used in additively manufactured molds. This study examines mechanical and thermal reclamation of spent furfuryl-resin sands obtained from binder-jetted molds (US-1) and conventionally manufactured molds (US-2) after ductile iron casting. Mechanical reclamation was conducted at rotor speeds of 280–560 rpm for 10–30 min, whereas thermal reclamation was performed at nominal temperatures of 600–1000 °C. Reclaim quality was assessed using loss on ignition (LOI), pH, collected dust content, and bending strength, with grain morphology used to support the interpretation of the results. At 560 rpm for 30 min, the mechanical-reclamation efficiency index (W_RK_) reached 31.5% for US-1 and 41.1% for US-2. At the highest nominal thermal-reclamation temperature after 30 min, LOI reached 0.14% and 0.29%, respectively. These results characterize each sand under the treatment and separation conditions applied to it. Different air-classification velocities—0.75 m/s for US-1 and 1.0 m/s for US-2—limit direct comparisons of collected dust, retained-matrix LOI, and the derived W_RK_ values. Together with differences in the initial sand characteristics, these classification conditions prevent differences between the systems from being attributed solely to the mold-manufacturing technology. Within each tested system, thermal reclamation achieved lower residual LOI, although selecting a process for practical use also requires an assessment of grain integrity and reuse performance.

## 1. Introduction

### 1.1. General Aspects of Spent Foundry Sand Reclamation

Reclamation of spent molding and core sands is defined as the treatment of used refractory materials to recover at least one component with properties comparable to those of the corresponding fresh material, thereby enabling its reuse in the production of casting molds and cores. In conventional mold- and core-making technologies based on silica sand and added binders, reclamation focuses primarily on recovering the sand matrix [1,2].

The production of castings in molds manufactured by additive techniques, most commonly binder jetting, is increasing [3,4,5]. The silica sand used in these processes generally has a considerably smaller mean grain size than that of the sands employed in conventional mold-making technologies. In binder jetting, self-hardening molds are frequently produced using furfuryl resin as the binder and an acidic curing system.

Although spent molding sands generated by conventional foundry processes can be treated effectively using mechanical, pneumatic, thermal, or combined reclamation methods, reclamation of spent sands obtained from additively manufactured molds has received comparatively little attention. To the best of the authors’ knowledge, no previous study has systematically compared the reclaimability of spent sand from binder-jetted molds after casting with that of an analogous conventionally prepared furfuryl-resin sand.

Regardless of the selected reclamation method, spent sands are most commonly processed through the following sequence of operations:

- Preliminary separation of mechanical contaminants, mainly metallic inclusions;
- Breakup of agglomerated sand after shakeout;
- Sieving and separation of grain-size ranges suitable for reclamation;
- Repeated separation of metallic contaminants;
- Secondary reclamation to remove residual spent binder coatings from sand grains;
- Removal of undesirable reclamation products by dedusting;
- Classification of sand grains according to the required size range and uniformity.

Treatment comprising the first four operations is referred to as primary reclamation [2]. Complete primary reclamation is required before binder coatings can be removed more intensively. At this stage, residual binder has not yet been sufficiently removed from the grains; the main effect is the breakup of large agglomerates and the preparation of the material for subsequent purification.

Secondary reclamation, together with primary reclamation, forms a complementary treatment system for removing residual binder coatings and technologically undesirable fractions from the sand matrix. More effective liberation of individual grains requires more intensive treatment than that used during primary reclamation.

**Figure 1:**
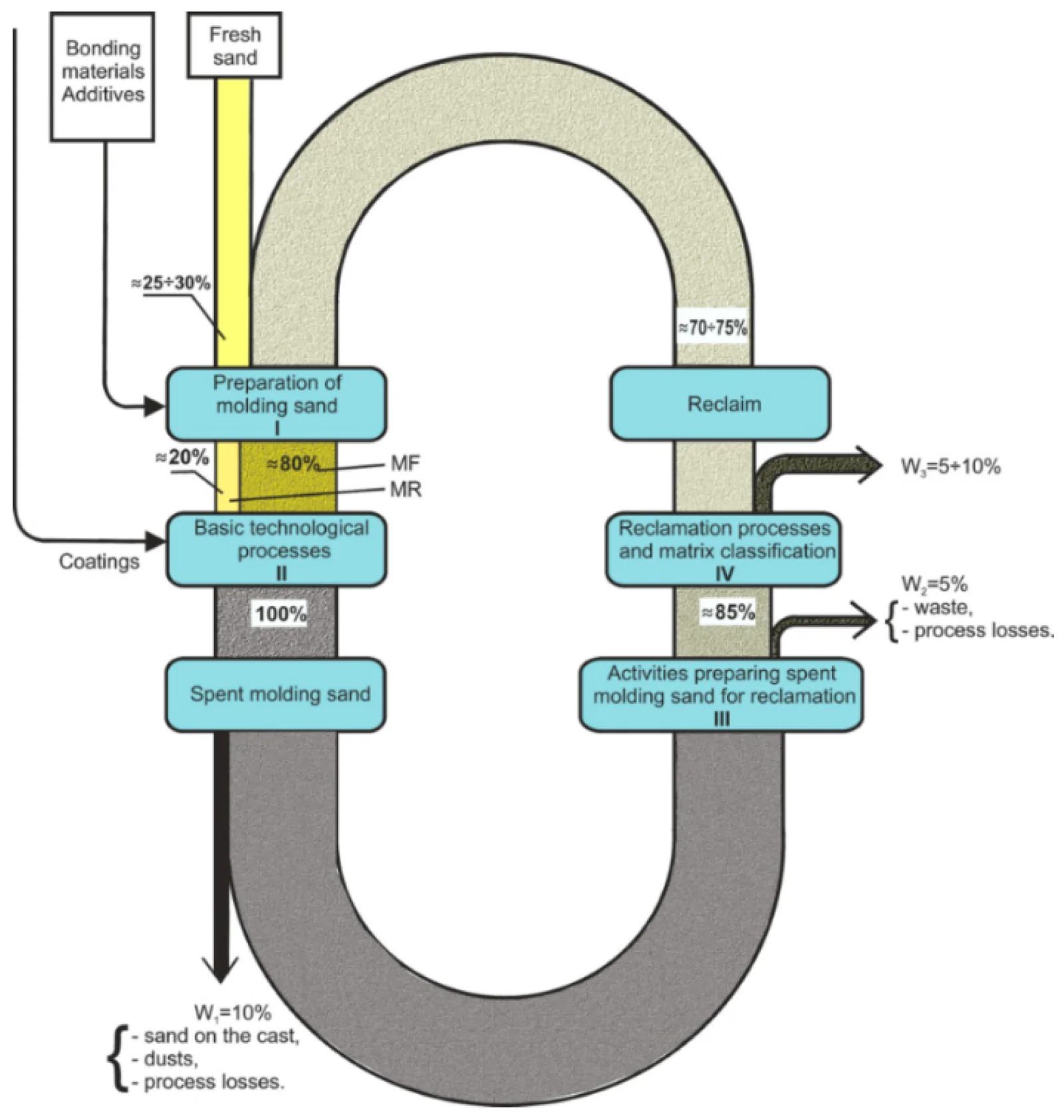
Circulation of a resin-bonded molding-sand matrix: I—molding and core sand preparation; II—mold and core production, pouring, casting solidification, cooling, and shakeout; III—spent-sand crushing and preparation for reclamation; IV—reclamation, dedusting, and separation of useful grains.

A typical matrix circulation loop in a foundry using chemically bonded molding sands is shown in Figure 1.

### 1.2. Reclamation Methods

Reclaimability describes how readily residual binder coatings can be removed from spent molding sands under specified treatment conditions [6,7,8]. It depends on binder–grain adhesion, matrix-grain size and morphology, binder content and physicochemical properties, chemical characteristics, molding technology, and the degree of thermal degradation.

Reclamation methods are classified as wet or dry. Wet reclamation is used mainly for green sands and water-soluble binder systems but requires large amounts of water, treatment of wastewater and sludge, and subsequent drying. The associated infrastructure requirements and costs limit its industrial use, while hydrophobic resin systems are generally unsuitable. Dry methods therefore predominate for chemically bonded sands.

#### 1.2.1. Mechanical and Pneumatic Reclamation

Dry mechanical reclamation offers broad applicability, relatively simple equipment, and lower implementation costs than more complex methods [2,8,9,10]. It is mainly used for self-hardening resin-bonded sands and selected inorganic binder systems, although binder removal may remain incomplete.

Cleaning involves rubbing between grains, abrasion against equipment surfaces, and crushing of binder coatings and bridges. Rubbing gradually thins the coating through mutual friction, whereas impacts can rapidly break binder bridges. Abrasion removes surface deposits through contact with moving or stationary equipment parts. Excessive treatment can also fracture the mineral grains. During mechanical and pneumatic reclamation, fine fractions and binder residues are separated using cyclones and air-extraction systems. Treatment intensity must therefore balance binder removal against the loss of useful matrix material.

#### 1.2.2. Thermal Reclamation

Thermal reclamation is primarily used to treat sands containing organic binders. It can also deactivate residual green-sand components in mixed systems and reduce the organic content of reclamation dust before disposal. High-temperature treatment decomposes thermally degradable binder components and improves matrix purification [6,7,11,12,13].

Its main advantage is extensive binder removal, which can produce a matrix approaching fresh-sand cleanliness and support its reuse in molds and cores. However, relatively high investment and operating costs and the potential need for off-gas treatment constrain implementation. Reported reclamation indices of approximately 80% for cold-box sands and 65% for hot-box sands illustrate that performance depends on the binder system and treatment conditions [1,6].

## 2. Materials and Methods

### 2.1. Research Objective

The aim of this study was to assess the feasibility of reclaiming the silica matrix from spent furfuryl-resin molding sands produced by additive binder jetting using a KOCEL AJS 300A system (KOCEL Intelligent Machinery Limited, Yinchuan, China). The test material consisted of spent sand obtained after pouring EN-GJS-500-7 ductile iron into the molds. The molding-sand-to-metal mass ratio was 1.6:1. An ASTM A 536-84 wedge casting was used as the test casting. For comparison, an identical casting was produced in a conventionally prepared furfuryl-resin mold. Shakeout was performed 30 min after pouring in both cases.

### 2.2. Binder Jetting Mold Production

The process begins with the preparation of the particulate material, which is distributed uniformly over the build platform by a recoater. The printhead then selectively deposits a liquid binder according to the geometry stored in the CAD file. The binder penetrates the powder bed and forms bridges between adjacent grains, creating a stable bonded structure.

After each layer is completed, the build platform is lowered by the prescribed layer height and the sequence is repeated until the entire object has been produced. After printing, unbound particulate material is removed and the resulting mold or core may undergo additional curing, depending on the material and process used [10,11,12,14,15].

**Figure 2:**
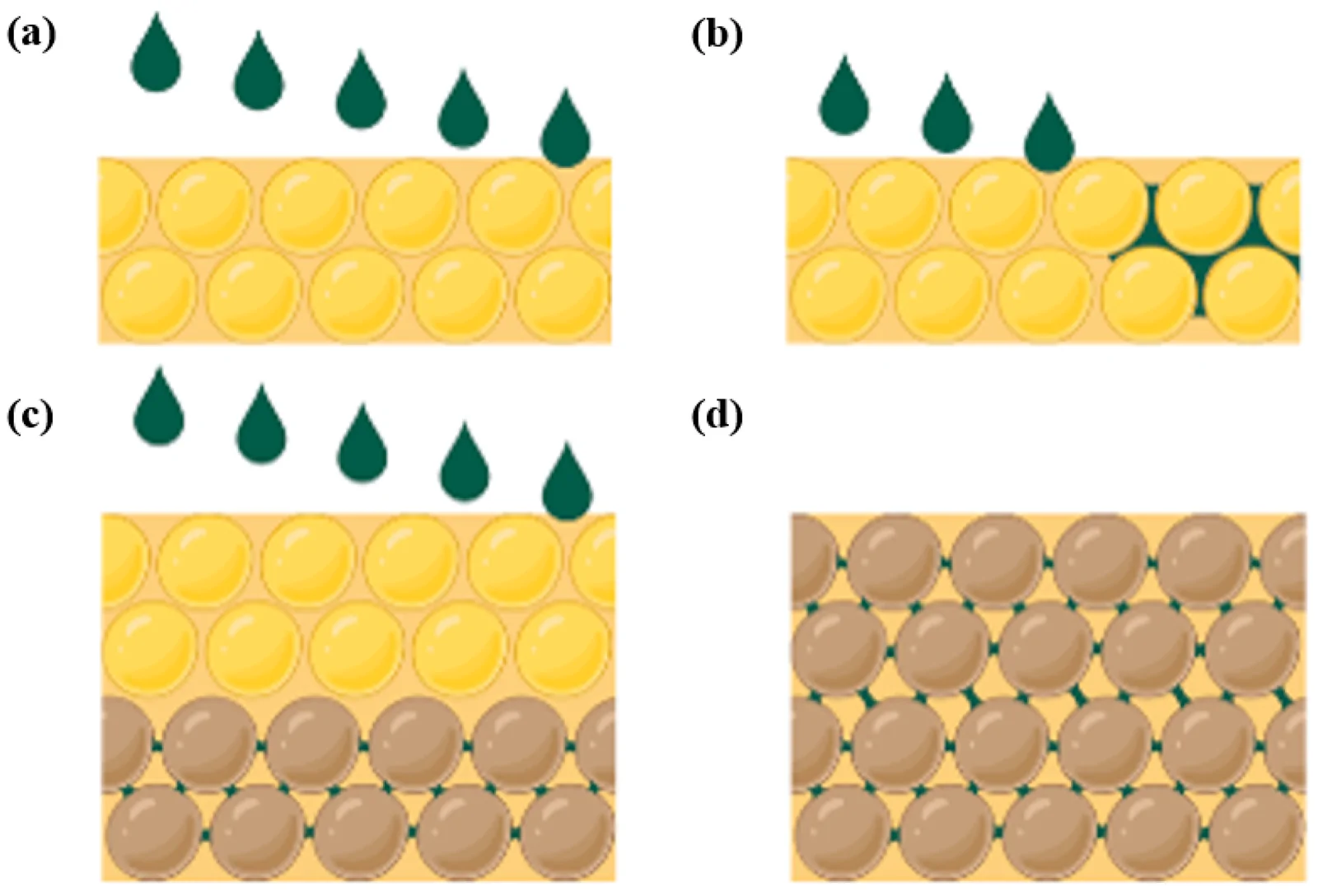
Schematic sequence of binder deposition in BJ: (**a**) droplets of liquid resin are deposited onto a sand layer already containing the curing agent; (**b**) the resin penetrates between sand grains; (**c**) a new layer of the ceramic matrix is deposited and selectively bound; (**d**) resin bridges form between adjacent matrix grains.

Figure 2 illustrates the mold-printing sequence in the BJ system. A layer of sand is spread over the build area by the recoater. Liquid binder is deposited by the printhead and penetrates the sand bed, forming bridges between the matrix grains. The recoater then applies the next sand layer, and the printhead selectively deposits binder. After each pass, the build platform is lowered by one layer thickness. This sequence is repeated until the target part is completed [5,13,16].

**Figure 3:**
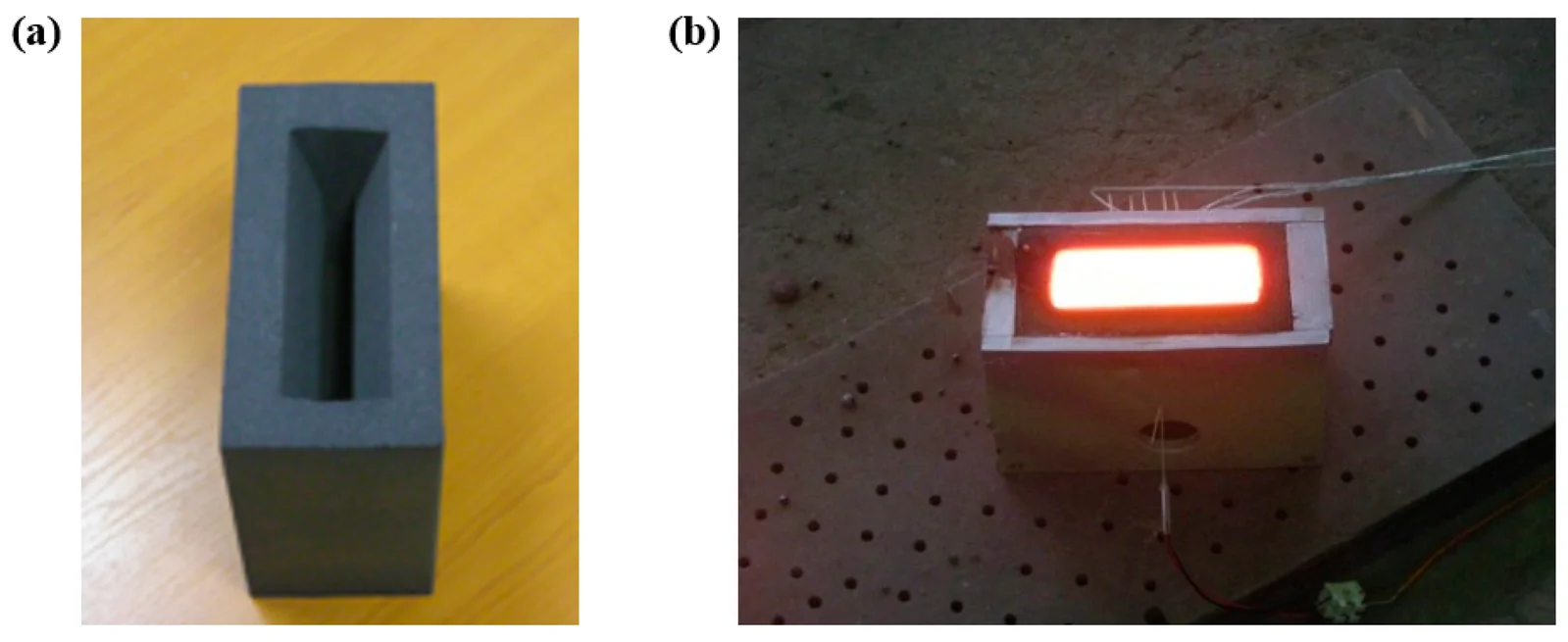
Mold for the ASTM A 536-84 wedge casting: (**a**) mold before pouring and (**b**) mold after pouring.

Figure 3 shows a mold produced by binder jetting. A dimensionally equivalent mold was also manufactured by the conventional method using silica sand with a chemically cured resin binder. The compositions of the molding mixtures used to prepare the binder-jetted (BJ) and conventional molds are provided in Table 1. For the BJ molding mixture, the formulation is prescribed by the equipment manufacturer, and the machine does not permit adjustment of the resin dosage delivered through the printhead. For the conventional molding mixture, the resin and hardener additions were deliberately set substantially above the levels recommended by the binder manufacturers. This formulation was selected to obtain comparable loss-on-ignition (LOI) values for the spent molding sands from both technologies after shakeout.

The same molten alloy was poured into the cured molds at the same temperature, and shakeout was performed after 30 min in both cases. The cooled spent sands from the two technologies were collected separately to evaluate reclamation performance. To obtain sufficient material for the experiments, 20 molds and castings were produced using each technology. This procedure yielded the two spent molding sands used in the reclamation experiments: US-1 from additive binder jetting and US-2 from conventional molding. Because the experiments were time- and labor-intensive, each reclamation condition was tested in a single process run. Three samples of the resulting reclaimed sands were collected for subsequent testing after each run. These samples represent within-run sampling rather than independent replicates of the reclamation process. Consequently, the reported variability does not quantify run-to-run reproducibility, and the results should be interpreted as descriptive comparisons under the conditions investigated.

### 2.3. Experimental Setup and Research Program

#### 2.3.1. Experimental Equipment

The experimental program comprised reclamation of prepared spent-sand samples, followed by characterization of the resulting reclaimed sands.

**Figure 4:**
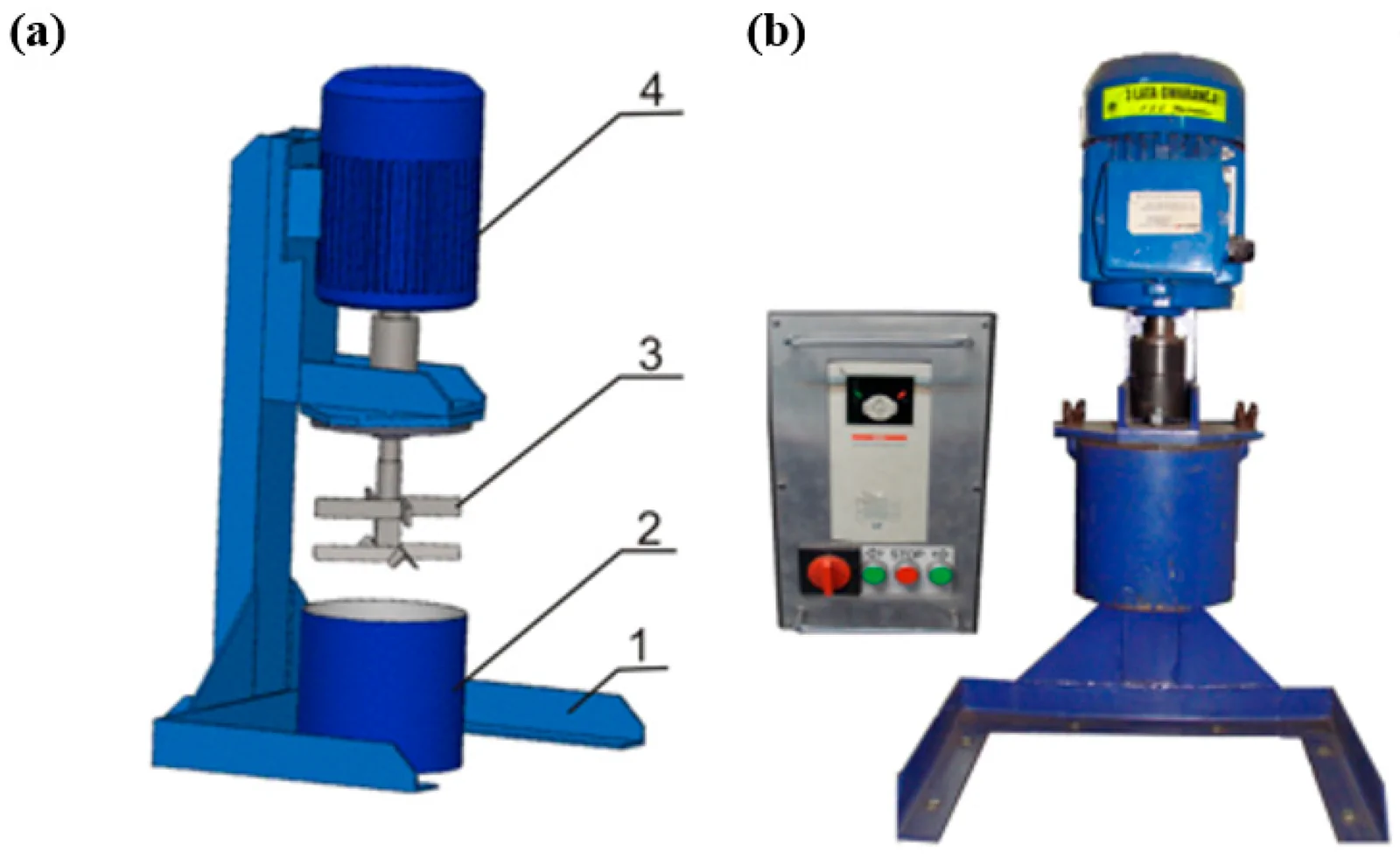
AT-2 rotary impact–abrasive test apparatus: (**a**) schematic view: 1—base, 2—container for the sand undergoing reclamation, 3—rotor of the impact–abrasive unit, 4—motor and (**b**) general view of the apparatus [17].

**Figure 5:**
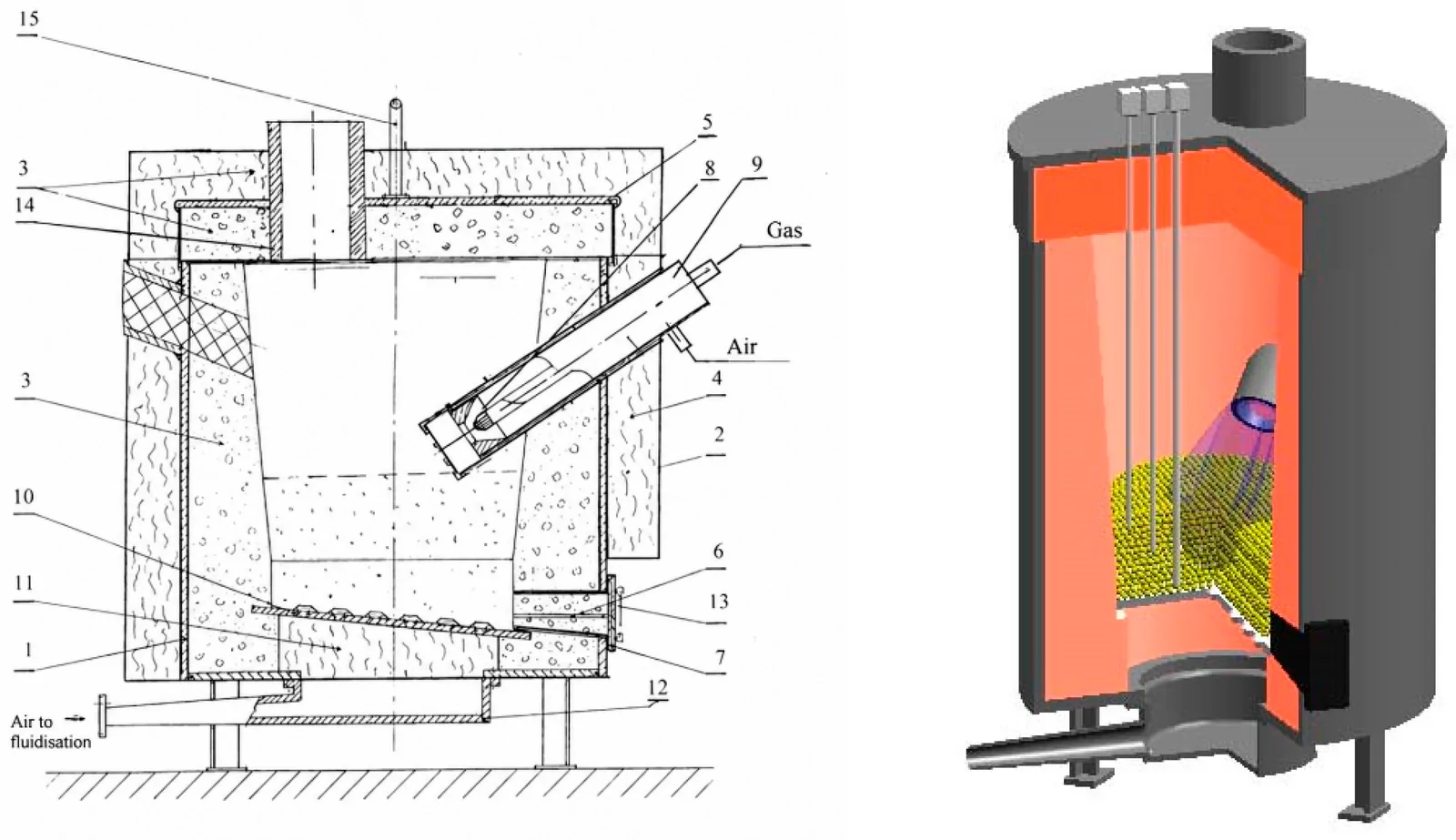
Experimental thermal reclaimer: schematic diagram and general view. 1—steel housing, 2—jacket, 3—refractory concrete, 4—ceramic-fiber insulation, 5—cover, 6—thermocouple port, 7—high-temperature seal, 8—burner, 9—burner housing, 10—perforated bottom, 11—ceramic wool, 12—air box, 13—discharge closure, 14—outlet nozzle, 15—suspension element [18].

Reclamation was performed using:

- The AT-2 rotary test apparatus operating through impact and abrasion (Faculty of Foundry Engineering, AGH University of Krakow—non-commercial prototype, Krakow, Poland) (Figure 4);
- An experimental thermal reclaimer (Faculty of Foundry Engineering, AGH University of Krakow—non-commercial prototype, Krakow, Poland) (Figure 5).

The AT-2 rotary impact–abrasive test apparatus (Figure 4) is a prototype device for laboratory studies of mechanical reclamation. It allows the rotor speed and treatment time to be varied. A 2 kg batch of spent sand was processed in each test.

After mechanical treatment in the AT-2 apparatus, each reclaimed batch was dedusted and pneumatically classified. The removed dust fraction consisted mainly of spent binder abraded from grain surfaces, together with some fine mineral particles. For conventionally produced molding sands, fractions below approximately 0.100 mm are commonly considered technologically undesirable, and an air-classification velocity of 1.0 m/s was used. Because the binder-jetted spent sand contained substantially finer matrix grains, preliminary tests showed that a lower classification velocity of 0.75 m/s was necessary to retain useful fine fractions and obtain a reclaimed grain-size distribution close to that of the fresh BJ sand. The reported dust content represents the fraction collected by pneumatic classification after mechanical reclamation, using air velocities of 0.75 m/s for US-1 and 1.0 m/s for US-2. This measurement depends on both the material produced or released during treatment and the extent of its separation under the selected classification conditions. It is therefore not an independent measurement of all fines generated during reclamation. Comparisons are made primarily within each sand system, for which the classification velocity was kept constant.

The experimental thermal reclaimer used in this study is shown in Figure 5. It is a batch thermal unit in which an air–gas mixture is burned in the furnace chamber above the surface of the spent sand. Batches of up to 5 kg were loaded into the reclaimer. A characteristic feature of the experimental unit, distinguishing it from conventional continuously fluidized thermal reclaimers, is the use of periodic fluidization to induce fountain-like mixing of the heated bed. Replacing continuous fluidization with cyclic air flow limits heat losses and excessive cooling of the bed by the fluidizing air. The fluidization system also enables improved control when technical-grade oxygen or oxygen–air mixtures of different compositions are supplied to the bed.

#### 2.3.2. Research Program

The experiments were designed to determine the potential for reclaiming spent sands from additive manufacturing and to provide a basis for further detailed studies.

Mechanical and thermal-reclamation tests were carried out using the experimental equipment described above.

**Figure 6:**
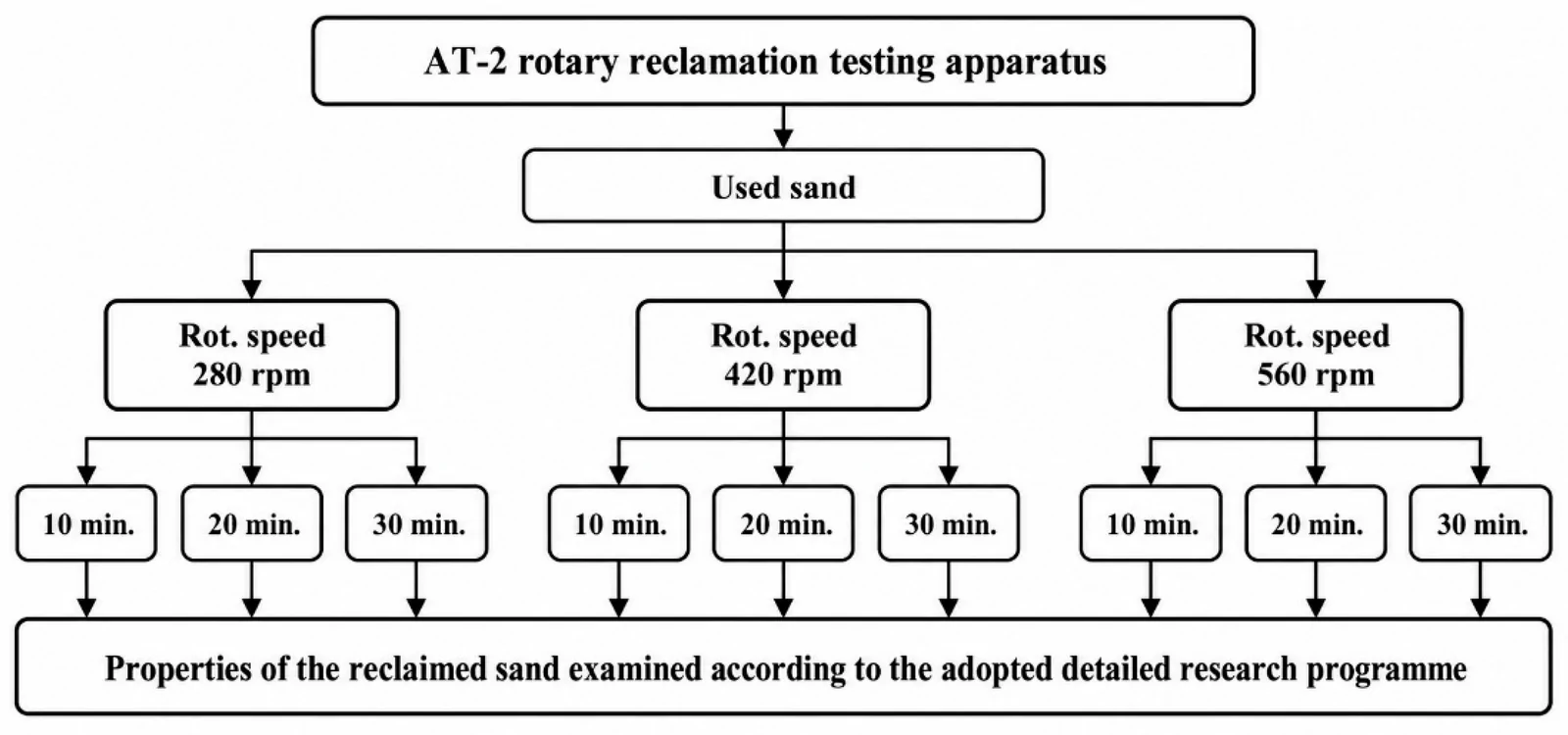
Experimental program for mechanical reclamation.

The mechanical-reclamation program is shown in Figure 6. The variables were the speed of the impact–abrasive rotor and the reclamation time.

**Figure 7:**
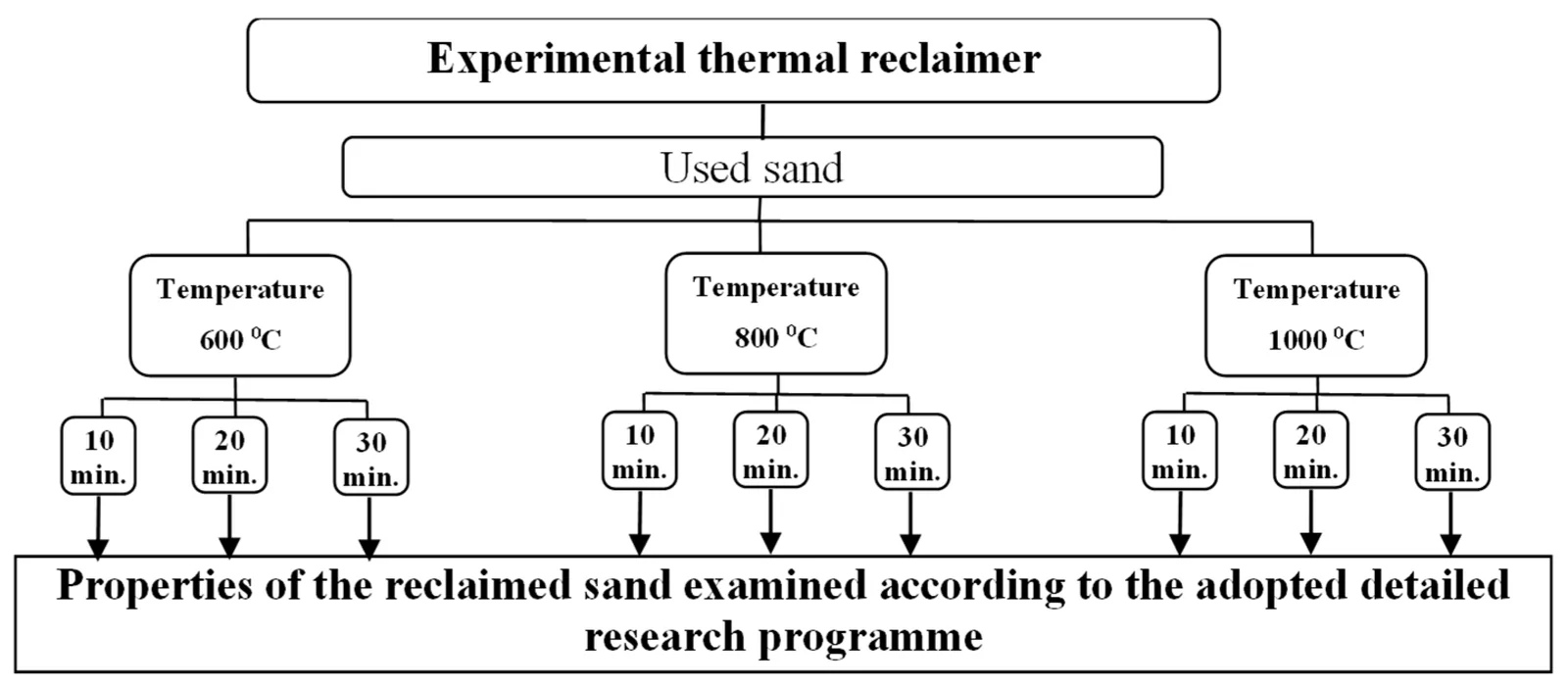
Experimental program for thermal reclamation.

The thermal-reclamation program is shown in Figure 7. The process variables were reclamation temperature and treatment time.

An additional variable in the comparative thermal-reclamation tests was the fluidization air velocity. A velocity of 1.0 m/s is commonly used for conventional molding sands. Preliminary tests showed that this value was too high for the finer binder-jetted spent sand because it removed matrix fractions below 0.100 mm that remain technologically useful in BJ applications. For this reason, a fluidization velocity of 0.75 m/s was adopted for US-1, whereas 1.0 m/s was used for US-2.

**Figure 8:**
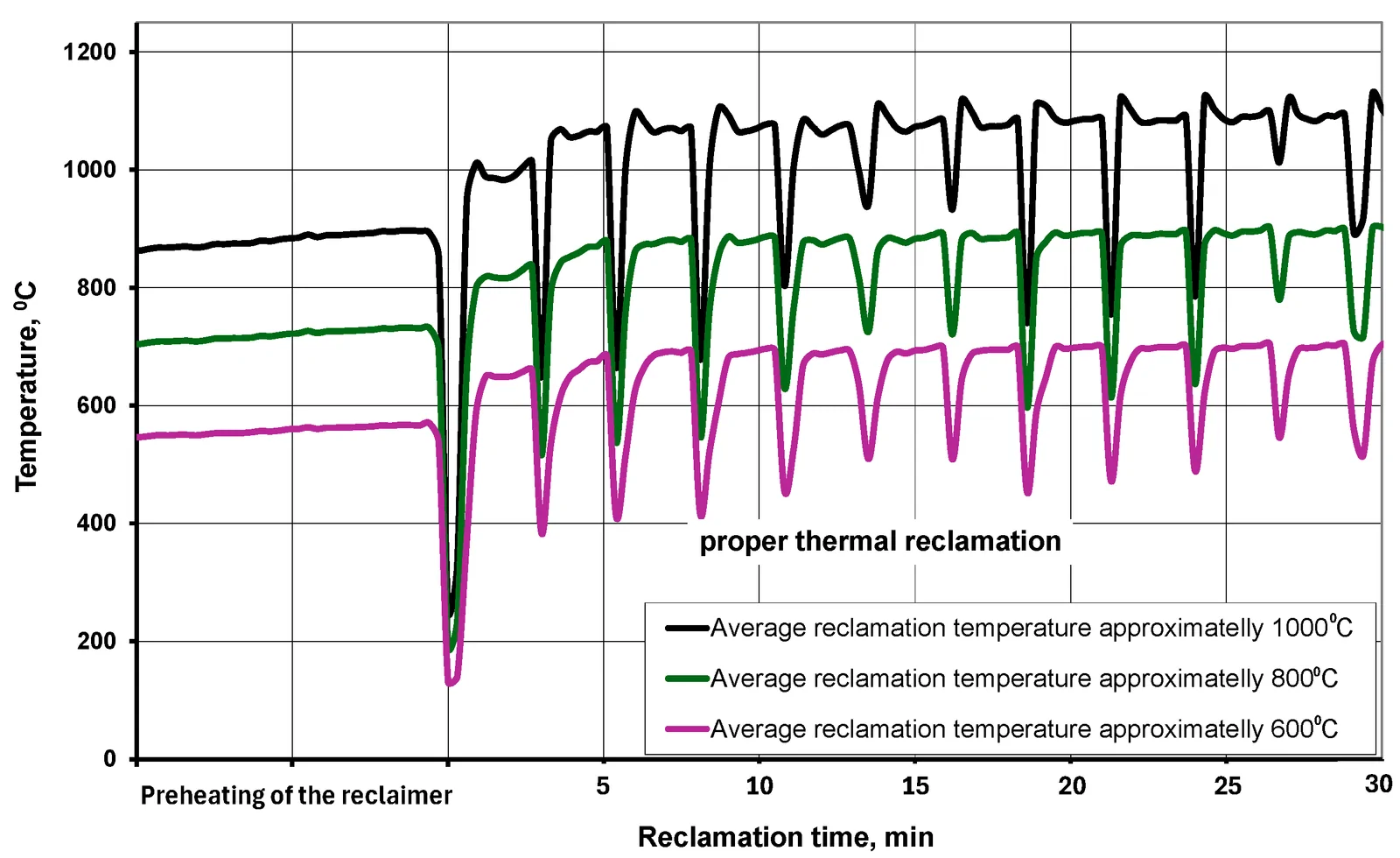
Temperature histories in the spent-sand bed during heating with periodic fluidization pulses. The fluidization pulse duration was 15 s. During the 30 min treatment, the bed underwent 11 heating stages and 11 fluidization pulses; the effective duration of a single heating stage was approximately 147 s (2.45 min).

Different temperatures during thermal reclamation were achieved by controlling the air–gas burner. The temperatures shown in Figure 7 represent the nominal process settings. The actual temperature histories recorded in the spent-sand bed at the different burner settings are shown in Figure 8.

The quality of the reclaimed sands obtained after mechanical and thermal treatment was evaluated using the following parameters:

- Loss on ignition (LOI) of the matrix after reclamation (percentage mass loss of a sand sample heated to 950 °C);
- Dust content in the reclaim (percentage mass loss of a sand sample purged with air at 1 m/s (US-2) or 0.75 m/s (US-1);
- LOI of the dust generated during mechanical reclamation;
- pH of the reclaimed matrix (determined by an electrochemical method in an aqueous suspension of the sand sample);
- Bending strength (Rgu) of molding sands prepared using matrices reclaimed at different treatment intensities, measured using rectangular specimens with dimensions of 22.4 × 22.4 × 170 mm;
- Particle-size distribution measured using an Analysette 22 NanoTec laser particle-size analyzer (FRITSCH GmbH, Idar-Oberstein, Germany).

Selected figures in the paper use non-zero y-axis origins to make variations within the measured ranges easier to distinguish. Readers should therefore assess the magnitude of differences using the numerical values and axis labels rather than the apparent relative heights of the bars.

## 3. Results and Discussion

### 3.1. Characteristics of the Spent Sands Before Reclamation

Two types of spent molding sands were subjected to reclamation:

- US-1 was obtained from the additive manufacturing process. The resin was based on furfuryl alcohol with phenolic and silane additives. At 25 °C, its viscosity was 20–60 mPa·s, density 1.1–1.2 g·cm^−3^, and pH 6–8. The catalyst was an acidic solution containing sulfonic acids and sulfuric acid [19].
- US-2 was obtained from conventional mold production. The binder system consisted of a urea–furfuryl resin and an aqueous p-toluenesulfonic acid solution. At 20 °C, the resin density was 1.175–1.185 g·cm^−3^, viscosity 0.030–0.045 Pa·s, free-formaldehyde content 0.12–0.14%, furfuryl-alcohol content 75 ± 1%, and nitrogen content 3.5 ± 0.5%. The catalyst density was 1.225 ± 0.01 g·cm^−3^ and its viscosity was approximately 15 mPa·s; the recommended storage temperature was above +5 °C.

The principal properties of the spent sands before secondary reclamation are listed in Table 2.

**Figure 9:**
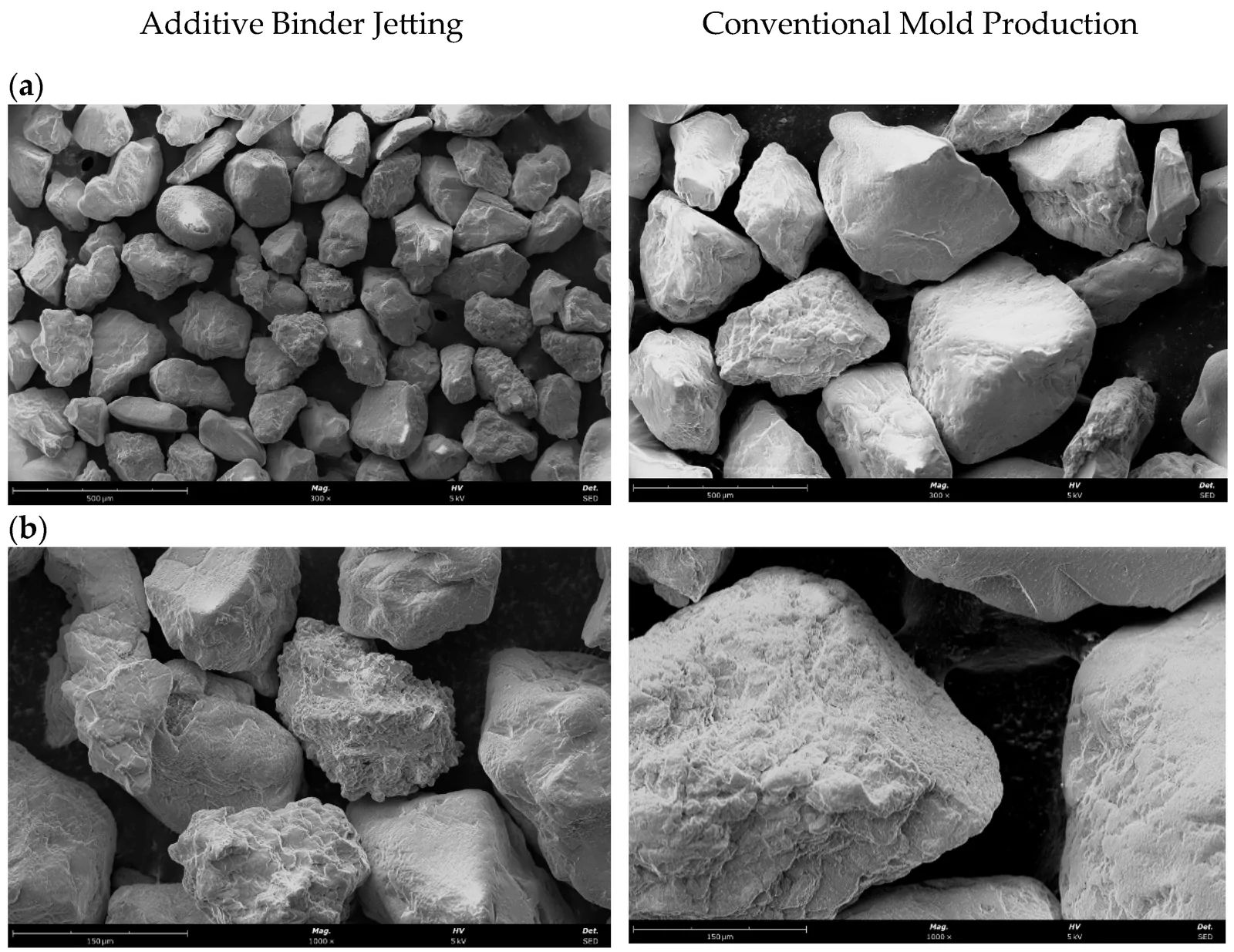
Surface morphology of the fresh silica sand grains used in additive binder jetting and conventional mold production. Magnification: (**a**) 300×; (**b**) 1000×.

**Figure 10:**
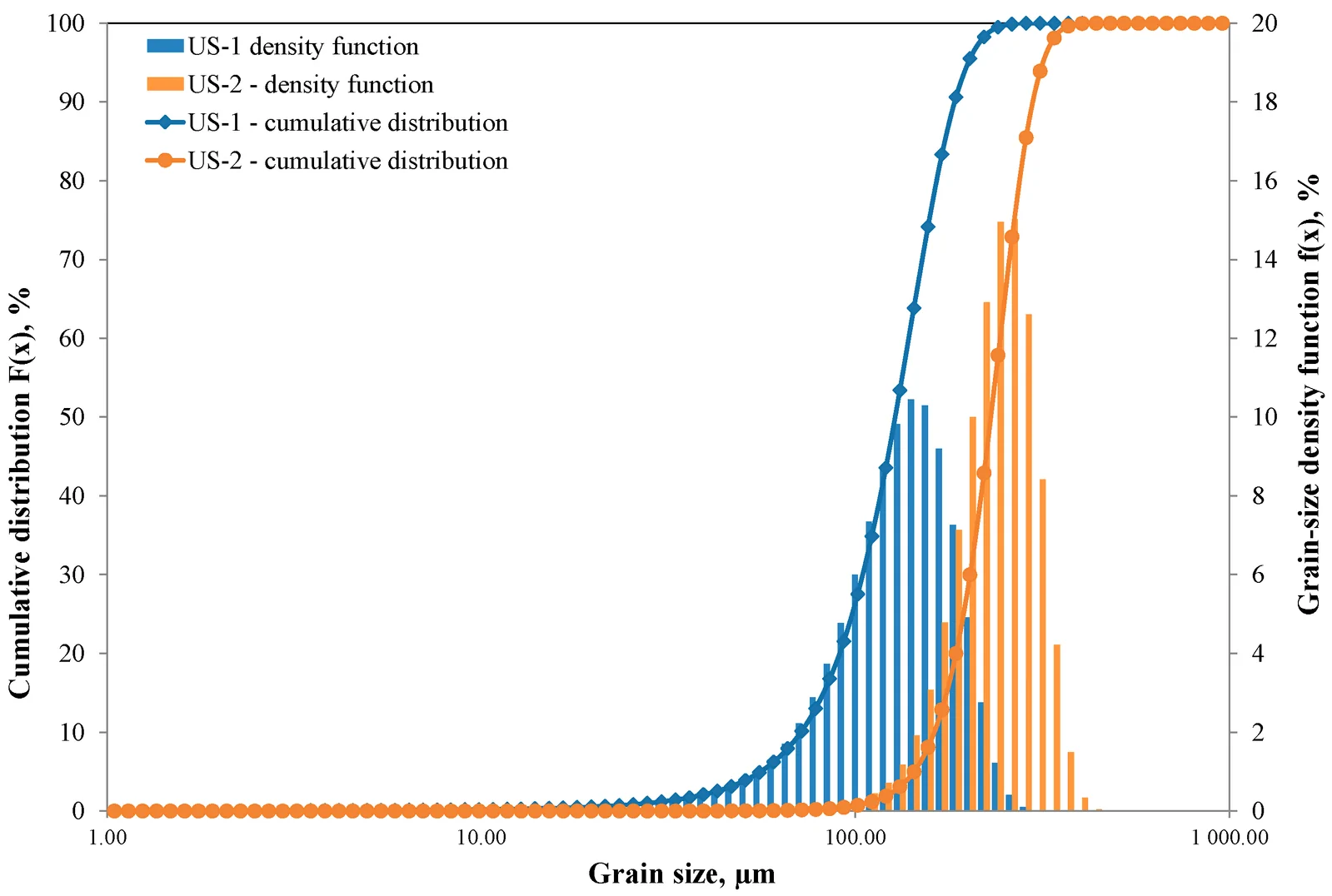
Particle-size distributions of spent molding sands US-1 and US-2 before reclamation. Columns indicate the percentage in each size class; curves show the cumulative distributions.

The data in Table 2 show a pronounced difference in the characteristic grain diameters of US-1 and US-2. The spent sand from additive manufacturing had a substantially smaller grain size, reflecting the use of much finer matrices in BJ than in conventional mold-making. Figure 9 compares SEM images of the fresh silica sand grains used to prepare molds by additive binder jetting and conventional molding. After pouring, shakeout, and preliminary crushing, the spent molding sands obtained from these molds were designated US-1 and US-2, respectively, and used in the reclamation experiments. The two spent sands represent different industrial material systems rather than a controlled comparison of mold-manufacturing technologies. Their geometric mean grain diameters are 0.125 mm for US-1 and 0.287 mm for US-2. The finer US-1 sand is expected to have a larger specific surface area per unit mass, which may affect binder distribution and the removal of residual coatings during mechanical reclamation. Figure 10 presents the particle-size distributions of the spent molding sands US-1 and US-2 after shakeout and preliminary crushing, before reclamation. The columns show the percentage in each size class, while the curves represent the cumulative distributions.

### 3.2. Loss on Ignition of the Reclaimed Sands

For spent sands bonded with organic resin, such as US-1 and US-2, LOI is a key indicator of reclaimed-sand quality because it reflects the amount of combustible binder residue remaining on the matrix. LOI was determined as the mass loss after oxidation for 2 h at 950 °C.

Both mechanical and thermal reclamation systematically reduced the LOI of the reclaimed sands, although the cleaning efficiency depended strongly on the origin of the spent sand and the treatment intensity. Before reclamation, LOI was 2.70% for US-1 and 2.85% for US-2, indicating a slightly higher initial content of residual organic material in the conventionally produced spent sand.

**Figure 11:**
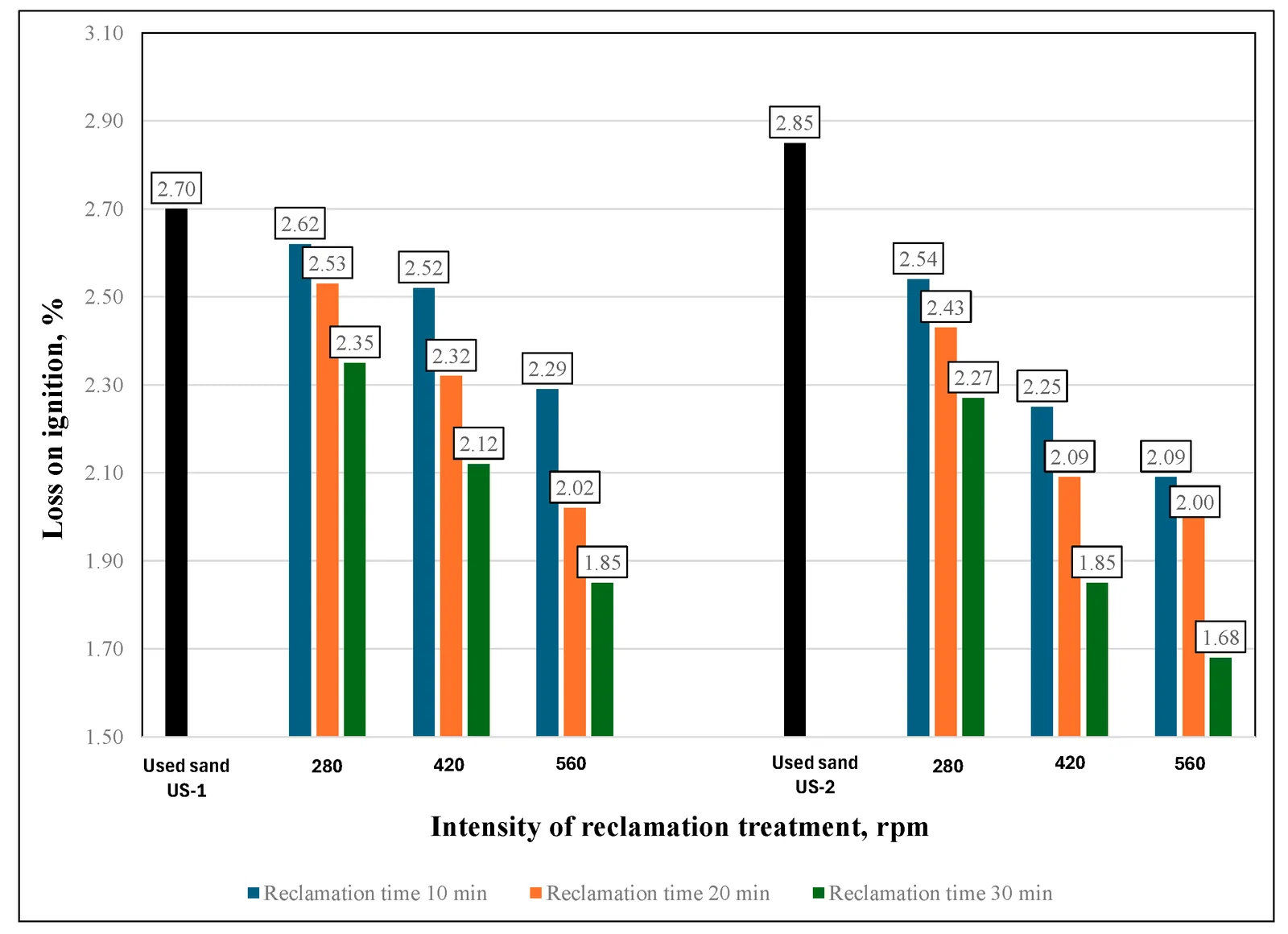
LOI of spent sands US-1 and US-2 and the corresponding reclaimed sands obtained at different mechanical-reclamation intensities. Note that the y-axis does not start at zero.

**Figure 12:**
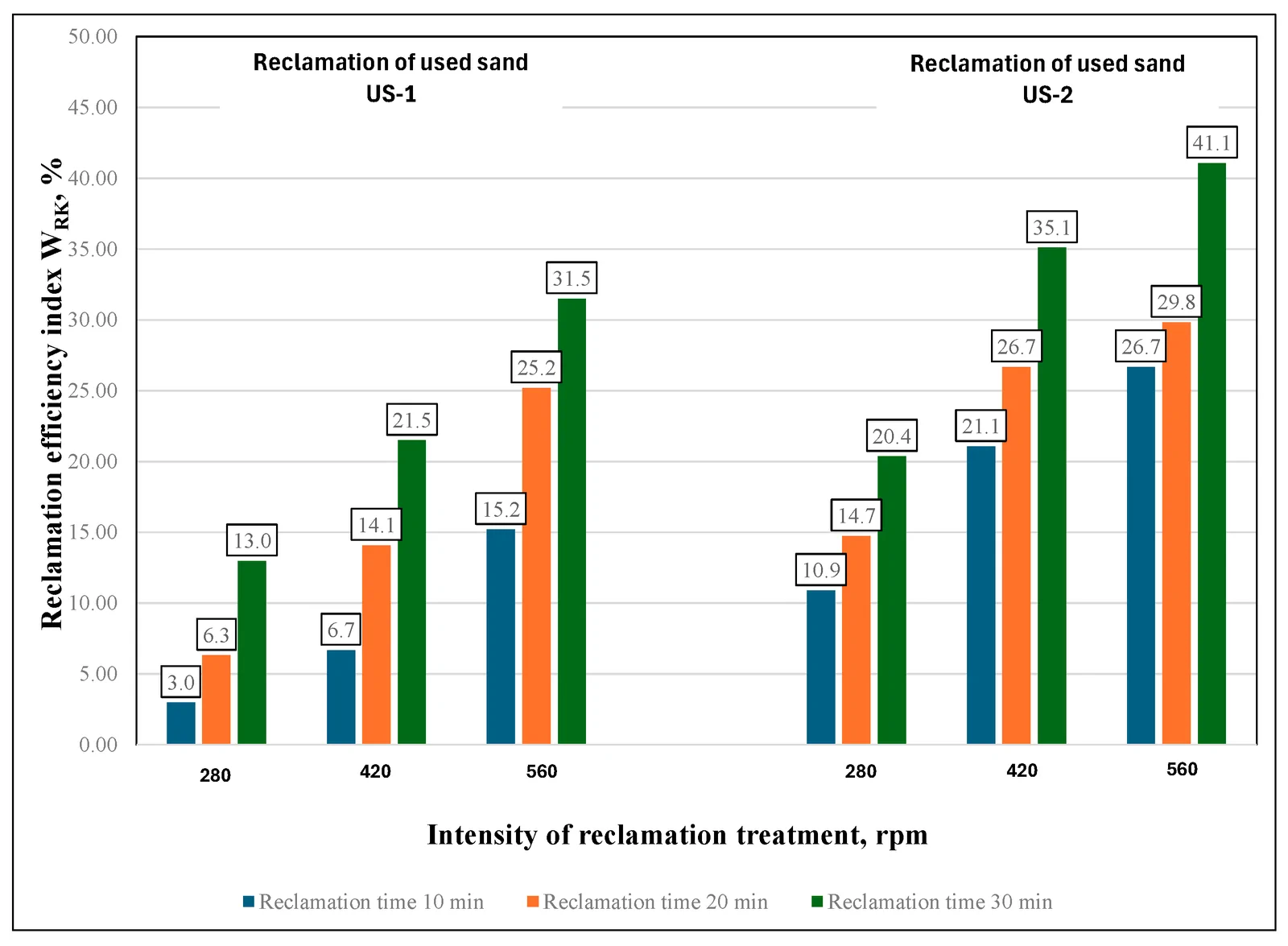
Reclamation efficiency index W_RK_ for US-1 and US-2 after mechanical reclamation at different treatment intensities.

During mechanical reclamation (Figure 11), increasing rotor speed and extending treatment time progressively reduced the LOI of both materials. For US-1, LOI decreased from 2.70% to 1.85% under the most intensive conditions, i.e., 560 rpm for 30 min. Under the same conditions, the LOI of US-2 decreased from 2.85% to 1.68%. Despite its slightly higher initial LOI, US-2 exhibited lower LOI values over almost the entire range of mechanical-reclamation conditions, showing that binder residues on the conventionally used matrix were removed more readily by impact–abrasive action. This trend is confirmed by the reclamation efficiency index W_RK_ shown in Figure 12. The W_RK_ index was calculated using the following equation:

(1) $$W_{RK}=\left(1-\frac{{LOI}_{R}}{{LOI}_{US}}\right)\cdot 100\%$$

where

W_RK_–reclamation efficiency index, %
LOI_US_–loss on ignition of the spent molding sand, %
LOI_R_–loss on ignition of the reclaimed sand, %

At 560 rpm for 30 min, the reclamation efficiency index reached 31.5% for US-1 and 41.1% for US-2. These values describe the relative reduction in LOI (LOI_R_) within each sand system. They do not establish an intrinsic difference in reclaimability attributable to mold-manufacturing technology, because the sands differ in grain size, morphology, and binder–catalyst chemistry.

**Figure 13:**
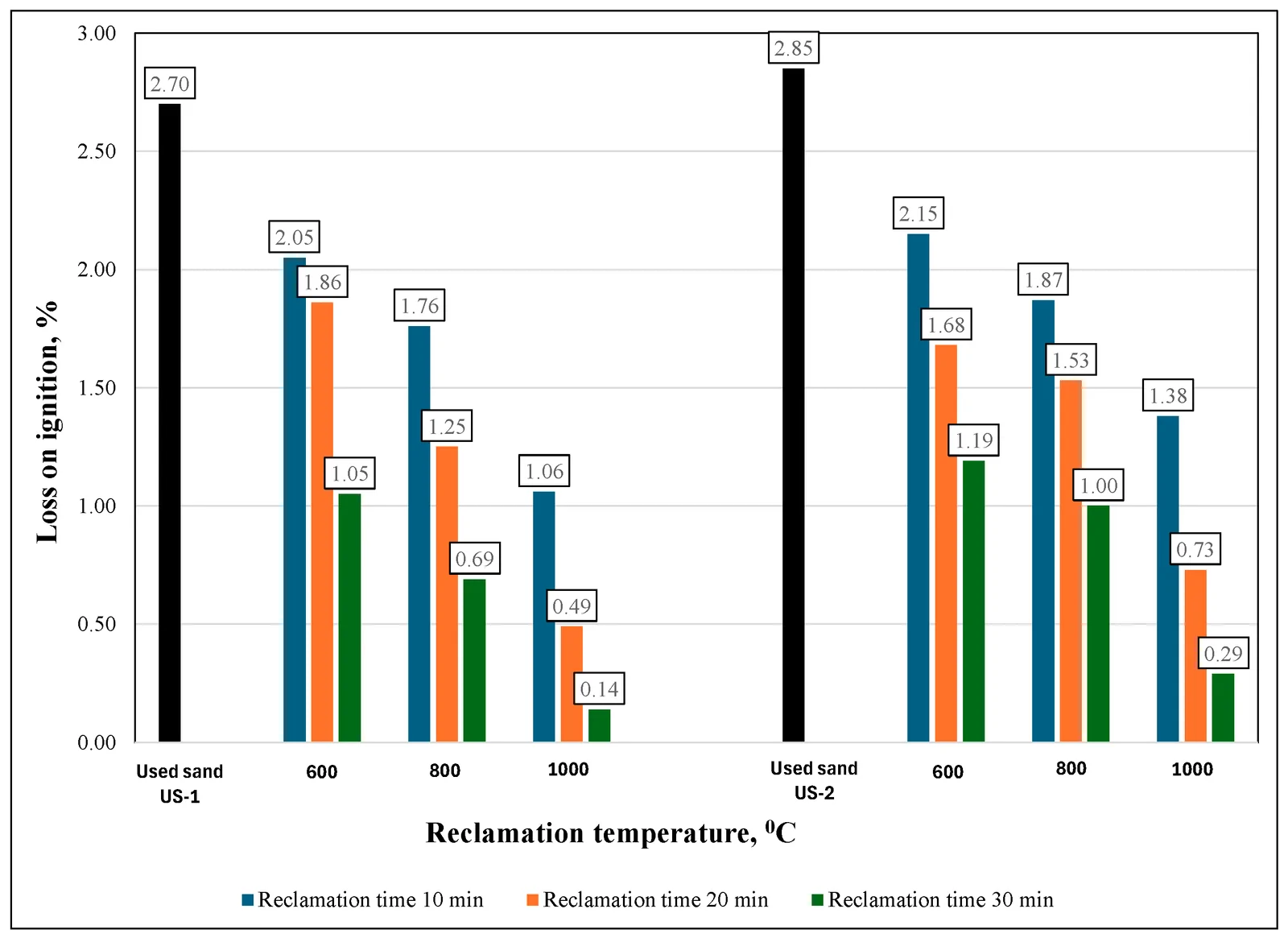
LOI of spent sands US-1 and US-2 and the corresponding reclaimed sands obtained at different thermal-reclamation temperatures.

**Figure 14:**
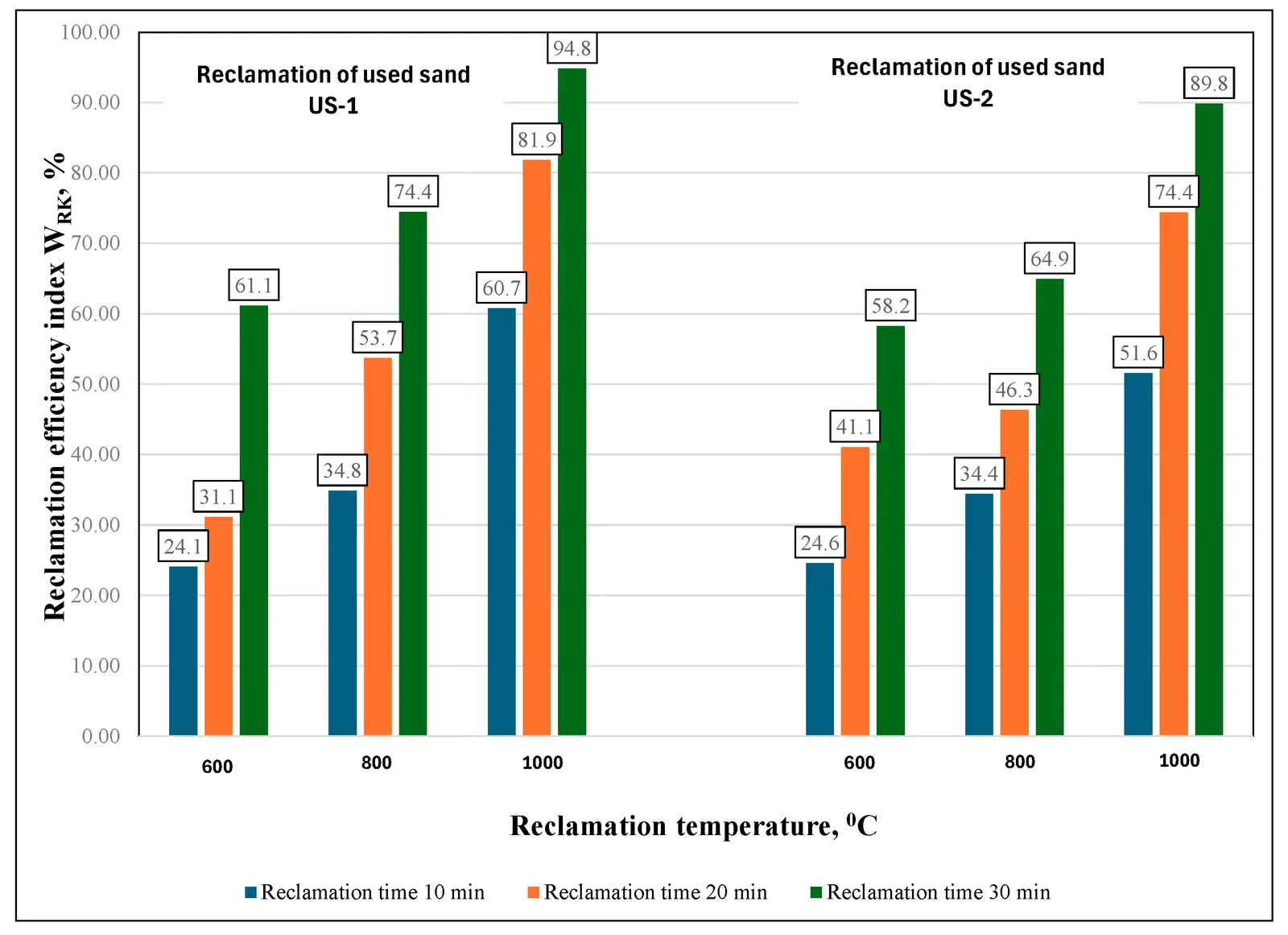
Reclamation efficiency index W_RK_ for US-1 and US-2 after thermal reclamation at different treatment conditions.

Thermal reclamation produced a substantially greater degree of matrix purification. As shown in Figure 13, increasing temperature and treatment time caused a pronounced decrease in LOI. Under the most intensive conditions, LOI (LOI_R_) fell to only 0.14% for US-1 and 0.29% for US-2. At the highest nominal temperature, the LOI (LOI_R_) values for US-1 after 10, 20, and 30 min were 1.06%, 0.49%, and 0.14%, respectively, whereas the corresponding values for US-2 were 1.38%, 0.73%, and 0.29%. The maximum W_RK_ values were 94.8% for US-1 and 89.8% for US-2 (Figure 14). These results demonstrate progressive removal of combustible residues within each sand system. Within both systems, thermal reclamation achieved lower residual LOI (LOI_R_) than mechanical treatment. However, differences in initial sand characteristics and applied air-classification conditions limit direct comparisons between US-1 and US-2. The reported values therefore characterize the tested material–process combinations and do not establish an intrinsic advantage of either mold-manufacturing technology.

The reclamation efficiency index W_RK_, calculated from the LOI of the reclaimed sands, describes the extent to which spent binder residues are removed from the matrix grains. Figure 13 and Figure 14 show W_RK_ values for US-1 and US-2 after mechanical and thermal reclamation, respectively.

### 3.3. pH of the Reclaimed Sands

**Figure 15:**
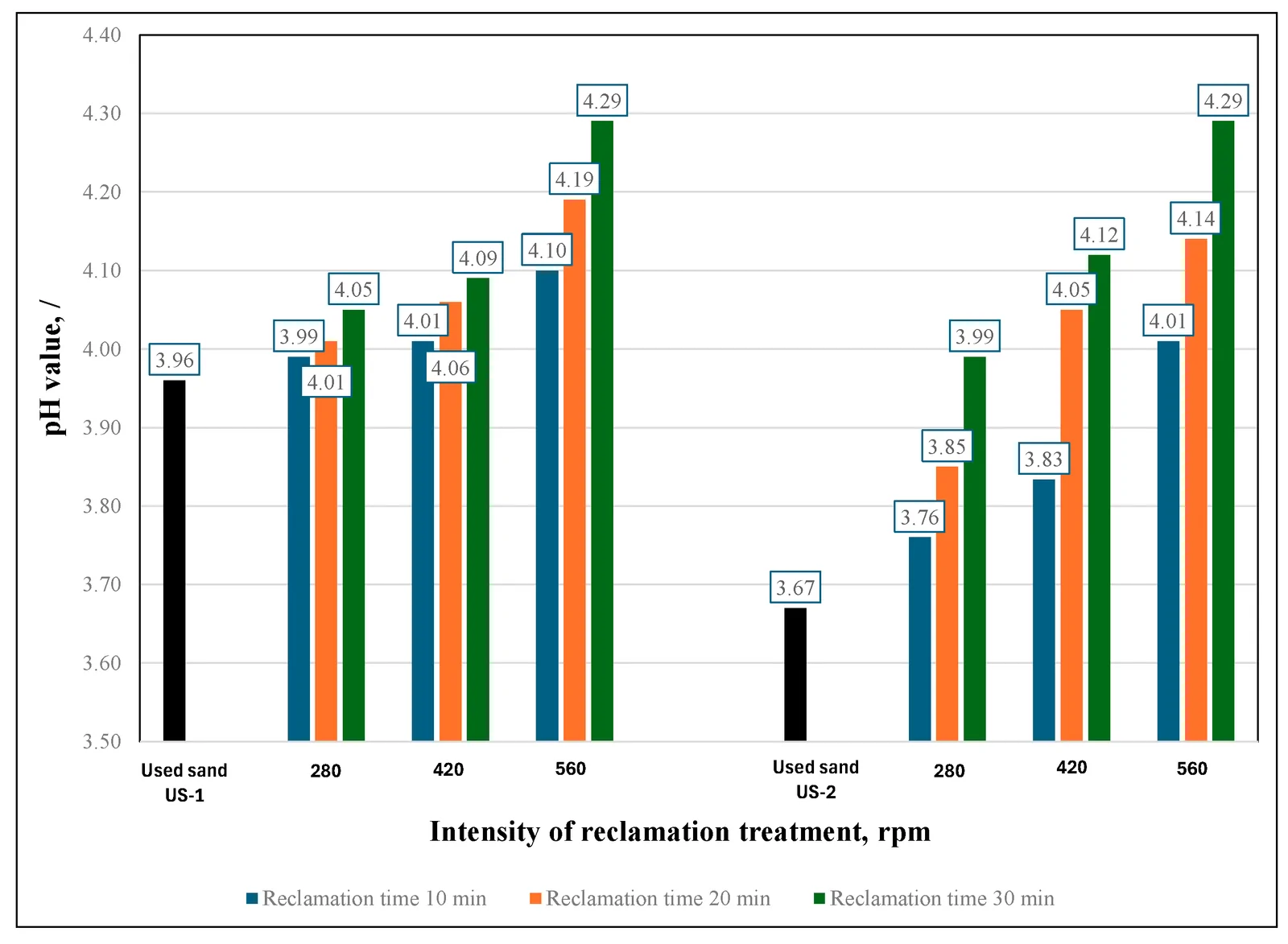
pH of US-1 and US-2 sands after mechanical reclamation at different intensities. Note that the y-axis does not start at zero.

**Figure 16:**
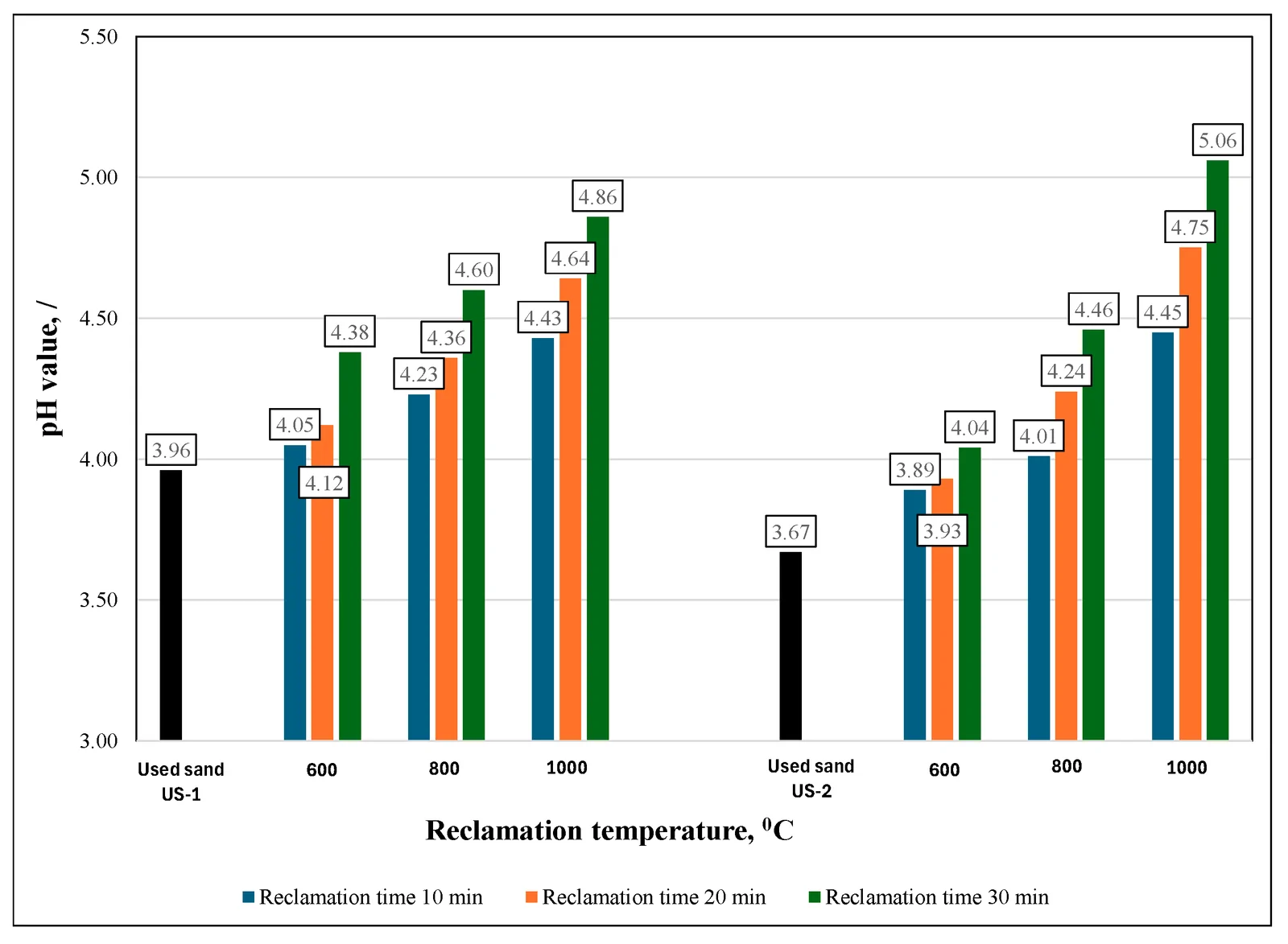
pH of US-1 and US-2 sands after thermal reclamation at different temperatures. Note that the y-axis does not start at zero.

The pH values of the reclaimed sands are presented in Figure 15 and Figure 16. For pH determination, a weighed sample of molding sand was mixed with distilled water at a sample-to-water mass ratio of 1:1. The suspension was shaken for 5 min and allowed to settle. The supernatant was then collected for pH measurement.

Increasing the intensity of both mechanical and thermal reclamation progressively reduced the acidity of the matrix. The initial pH was 3.96 for US-1 and 3.67 for US-2. The difference is attributable to the different resin-catalyst systems and the amount of residual acidic curing agent.

During mechanical reclamation (Figure 15), pH increased with rotor speed and treatment time, reaching approximately 4.29 under the most intensive conditions. This increase resulted from partial removal of binder residues and acidic catalyst components from grain surfaces and was consistent with the decrease in LOI. Nevertheless, both reclaimed sands remained acidic, confirming that mechanical treatment did not completely purify the matrix.

A larger change in pH was observed after thermal reclamation (Figure 16). With increasing temperature and treatment time, pH increased to 4.86 for US-1 and 5.06 for US-2, indicating more effective decomposition and removal of acidic binder and catalyst residues.

The pH trends were generally consistent with the LOI results: a decrease in residual organic matter was accompanied by an increase in pH. The relationship was not proportional, however, because LOI represents the total amount of combustible material, whereas pH is governed mainly by soluble acidic species. The two parameters should therefore be treated as complementary indicators of reclaimed-sand purification.

### 3.4. Particle-Size Distribution

Following mechanical reclamation and final classification, the reclaimed sands were subjected to particle-size distribution analysis to assess the extent of sand-grain fragmentation. The values presented in Table 3 show a progressive decrease in both the arithmetic (d_a_) and geometric (d_g_) mean grain diameters with increasing reclamation time and rotational speed. At 280 rpm, the mean diameters gradually approach those of the corresponding fresh sands, remaining slightly higher after 30 min. At 420 and 560 rpm, both mean diameters fall below the fresh-sand values after 30 min. The largest reductions occur at 560 rpm for 30 min, where d_a_ reaches 0.1266 mm for US-1 and 0.3043 mm for US-2, compared with fresh-sand values of 0.1378 and 0.3172 mm, respectively. This pattern is consistent with the removal of residual binder coatings and the breakdown of agglomerates during reclamation, followed by an increasing contribution from grain fragmentation under more intensive treatment. However, mean grain diameters alone cannot distinguish these mechanisms, and the measurements also reflect the effect of final classification on the particle-size distribution of the retained material. Consequently, values below those of fresh sand suggest possible grain fragmentation but do not independently confirm it. Because US-1 and US-2 differ substantially in their initial grain sizes, their responses to reclamation should be compared relative to their respective reference sands rather than solely in terms of absolute diameters.

### 3.5. Amount and Loss on Ignition of Dust Generated During Reclamation

Dust is an important by-product of mechanical reclamation of spent molding sands. It forms as residual binder layers are abraded and crushed from matrix-grain surfaces. The amount of dust can therefore serve as an additional indicator of cleaning intensity, although excessive dust generation may also indicate degradation of the mineral matrix.

Dust quantity was determined only after mechanical reclamation and subsequent pneumatic classification. During thermal reclamation, organic binder residues undergo decomposition and oxidation; however, mineral fines may also be produced or released through thermally induced cracking, chipping, or particle interactions. Dust generation and losses of mineral fines during thermal treatment were not quantified in this study. The reported dust measurements therefore cannot be used to compare dust generation between mechanical and thermal reclamation.

The different classification velocities reflected the different grain-size distributions of the two matrices. US-1, from additive manufacturing, had a much finer main fraction (0.10/0.071/0.16 mm), whereas US-2 had a main fraction of 0.20/0.16/0.32 mm. Accordingly, a lower dedusting velocity of 0.75 m/s was used for US-1, compared with 1.0 m/s for US-2. These values were selected to separate binder-abrasion products while limiting the loss of technologically useful matrix grains, particularly from the fine-grained binder-jetted material.

**Figure 17:**
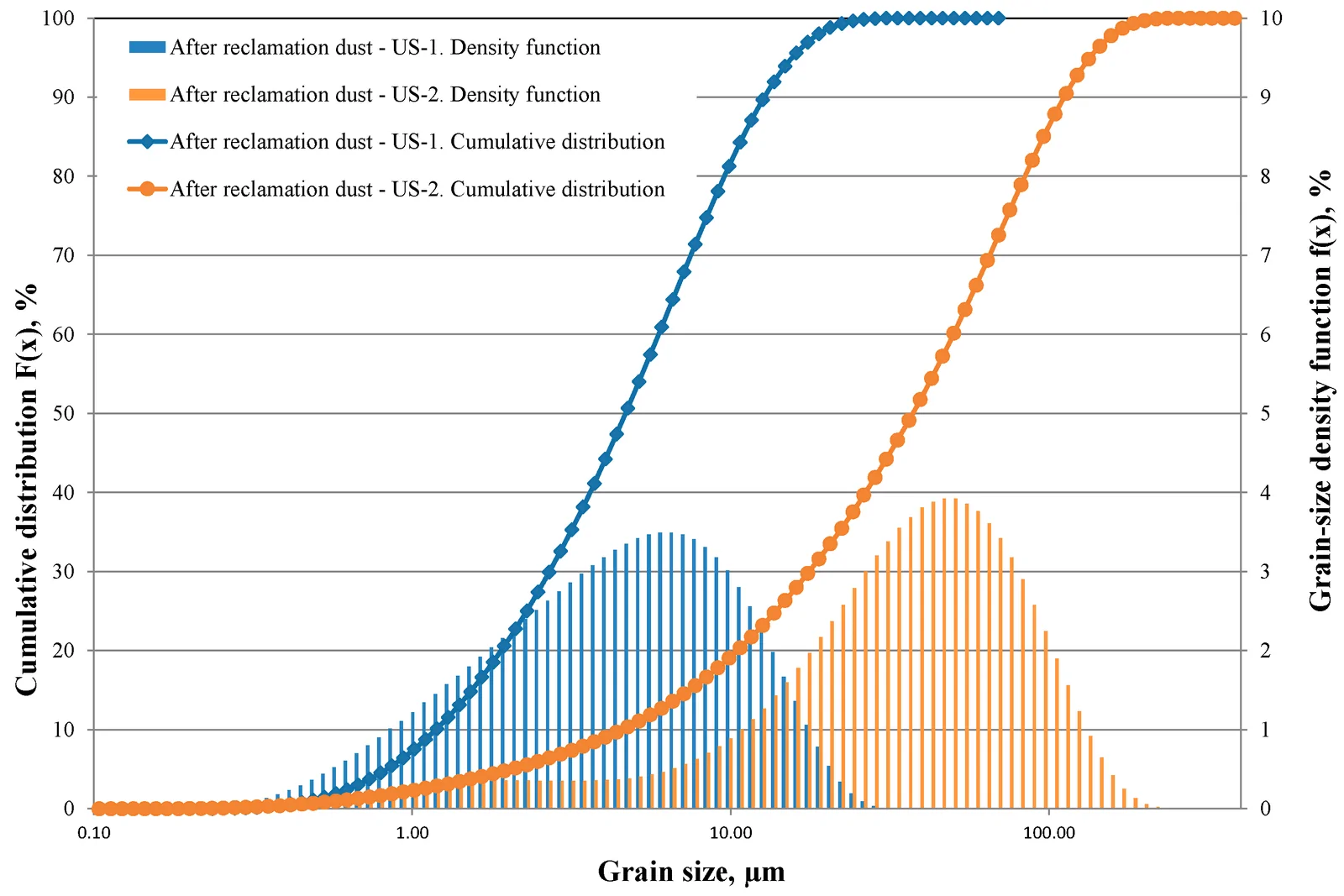
Particle-size characteristics of dust samples with the highest LOI values collected after reclamation.

The particle-size distributions in Figure 17 show that dust generated during reclamation of US-1 was much finer than that obtained from US-2. The density distribution peaked at approximately 5–6 µm for US-1 and approximately 45–55 µm for US-2. The cumulative distributions indicate estimated median particle diameters (D50) of approximately 5 µm and 40 µm, respectively. Approximately 90% of the particles were smaller than 13–15 µm for US-1 and approximately 100 µm for US-2. These differences reflect both the different initial matrix-grain sizes and the different pneumatic-classification velocities.

**Figure 18:**
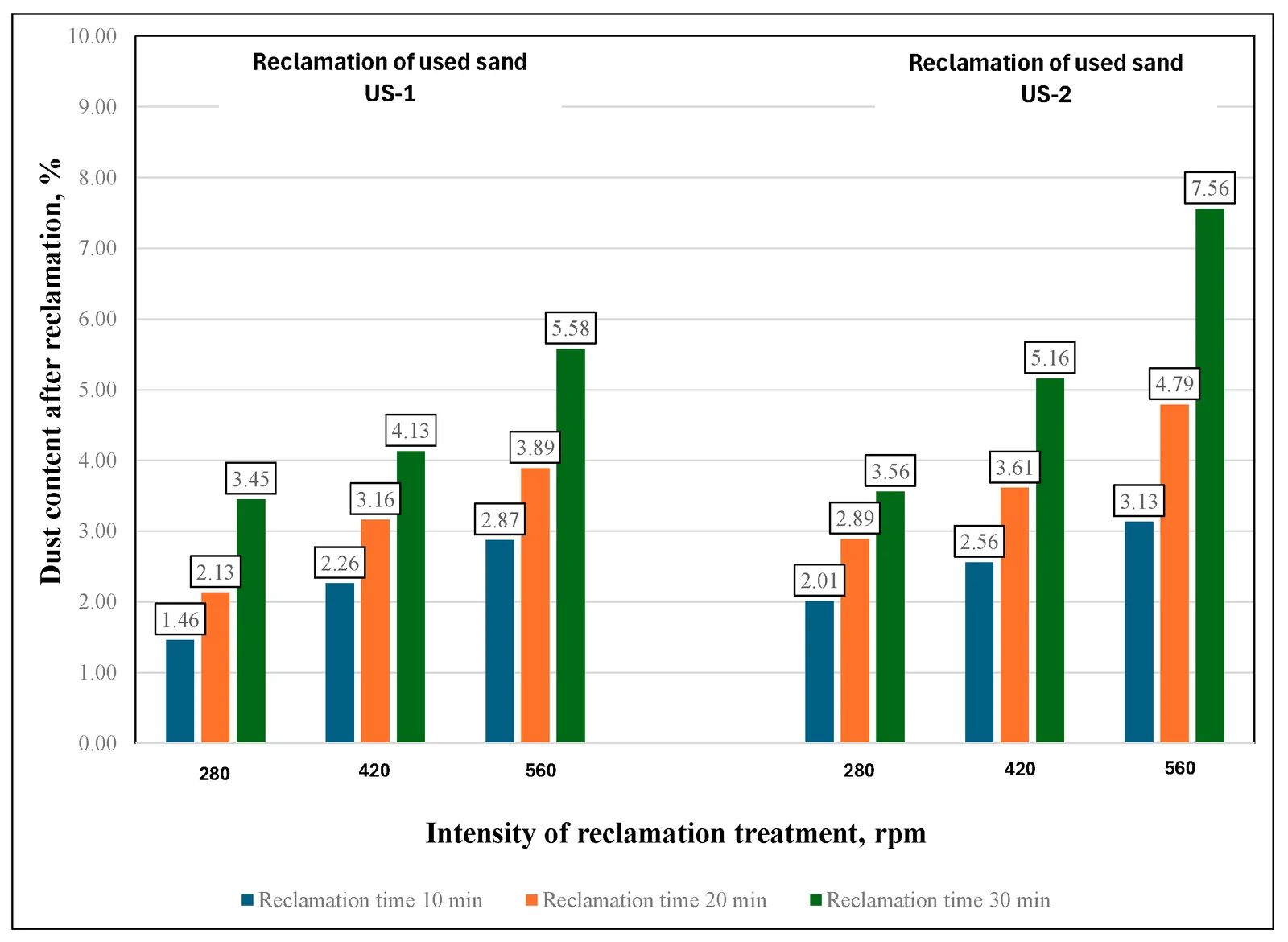
Collected dust fraction after mechanical reclamation and subsequent pneumatic classification of US-1 at 0.75 m/s and US-2 at 1.0 m/s. Differences between the systems also reflect the different classification conditions.

As shown in Figure 18, the collected dust fraction increased with rotor speed and reclamation time within each sand system. It ranged from 1.46% to 5.58% for US-1 at a classification velocity of 0.75 m/s and from 2.01% to 7.56% for US-2 at 1.0 m/s. These ranges characterize the combined effects of mechanical treatment and subsequent pneumatic classification under the conditions used for each material. The difference between these absolute values cannot establish which sand intrinsically generates more dust, because the effects of initial grain-size distribution and classification velocity were not independently evaluated.

**Figure 19:**
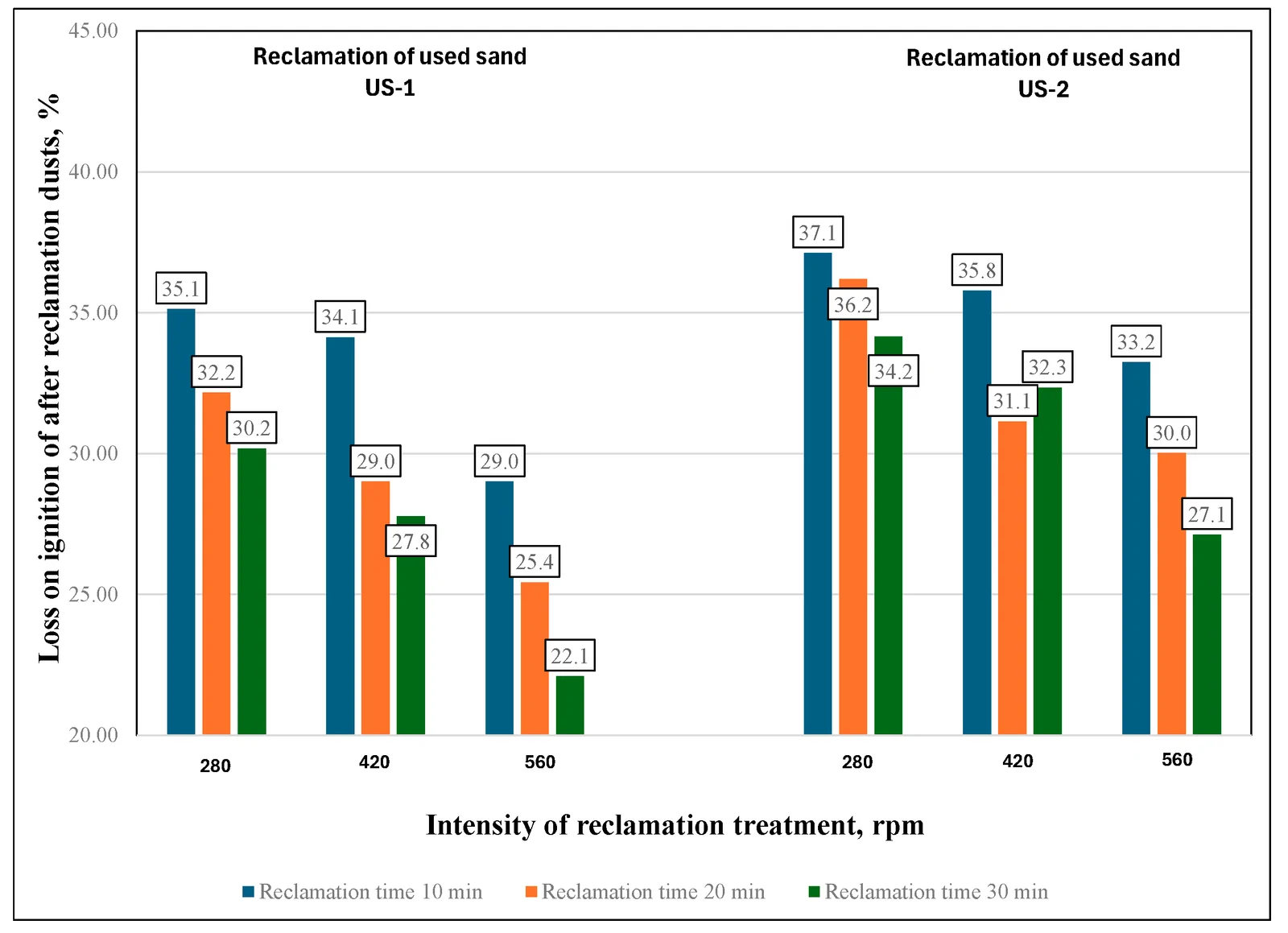
LOI of dust generated during mechanical reclamation of spent sands US-1 and US-2. Note that the y-axis does not start at zero.

As shown in Figure 19, increasing reclamation intensity simultaneously reduced the LOI of the generated dust. For US-1, dust LOI decreased from 35.1% to 22.1%, whereas for US-2 it decreased from 37.1% to 27.1%. This trend is consistent with an increasing proportion of non-combustible material relative to combustible residues in the collected dust.

The simultaneous increase in collected dust content and decrease in dust LOI is consistent with an increasing proportion of non-combustible material in the collected fraction. However, these measurements do not distinguish newly fractured grains from pre-existing mineral fines released during cleaning and subsequently separated by classification. Grain abrasion or fragmentation may contribute but cannot be confirmed from the dust data alone. This interpretation is consistent with the limitations of the particle-size analysis discussed in Section 3.4.

### 3.6. Bending Strength of Molding Sands Prepared with Reclaimed Matrix

Bending strength is one of the key technological properties of molding and core sands. It determines the resistance of molds and cores to damage during production, handling, assembly, and pouring. Measuring bending strength therefore provides a practical assessment of whether reclaimed matrix can be reused without an unacceptable deterioration in molding-sand performance.

Tests were performed on molding sands prepared with fresh silica sand and with matrices obtained by mechanical (Table 4) and thermal (Table 5) reclamation. For mechanical reclamation, mixtures containing either 100% reclaimed matrix or 50% reclaimed matrix were evaluated. The reference strengths obtained with fresh silica sand differed between the two binder systems: 5.61 MPa for US-1 and 3.89 MPa for US-2. The reclaimed-sand results should therefore be interpreted relative to their corresponding fresh-sand reference values.

When 100% mechanically reclaimed matrix was used, increasing reclamation intensity and treatment time generally increased bending strength. For US-1, bending strength (Rgu), measured using rectangular specimens with dimensions of 22.4 × 22.4 × 170 mm, increased from 1.13 MPa after the least intensive treatment to 3.69 MPa after 30 min at the highest rotor speed. For US-2, the corresponding increase was from 0.89 MPa to 2.58 MPa. More intensive cleaning therefore improved bonding between the reclaimed grain surfaces and the fresh binder, although the strength remained below that of the fresh-sand reference mixtures.

For mixtures containing 50% mechanically reclaimed matrix and 50% fresh sand, bending strength should be assessed relative to the corresponding fresh-sand reference for each system. The maximum strength of US-1 was 4.69 MPa, obtained after reclamation at 560 rpm for 30 min, representing 83.6% of its reference strength of 5.61 MPa. For US-2, the maximum was 3.45 MPa, obtained at 420 rpm for 30 min, representing 88.7% of its reference strength of 3.89 MPa. Thus, US-1 exhibited the higher absolute maximum strength, whereas US-2 retained a larger proportion of its own lower reference strength. These percentages describe within-system strength retention and do not establish the superiority of either mold-manufacturing technology. For US-2, increasing the rotor speed from 420 to 560 rpm at 30 min reduced the measured strength from 3.45 to 3.05 MPa; the mechanism underlying this reduction was not isolated.

For mixtures containing 100% reclaimed matrix, the highest measured bending strengths after thermal reclamation were 4.43 MPa for US-1 and 3.34 MPa for US-2, compared with 3.69 and 2.58 MPa, respectively, after mechanical reclamation. The values obtained with thermally reclaimed matrix corresponded to approximately 79% and 86% of the respective fresh-sand reference strengths. Together with the lower residual LOI, these results are consistent with a beneficial contribution from binder-residue removal. Nevertheless, bending strength reflects the combined effects of residual coatings, grain characteristics, packing, and bonding. The present measurements do not isolate these contributions or exclude simultaneous thermal damage to the matrix.

For mixtures containing 50% thermally reclaimed matrix and 50% fresh sand, the highest bending strengths were 4.14 MPa for US-1 and 3.12 MPa for US-2, both obtained after reclamation at 1000 °C for 30 min (Table 5). These values corresponded to 73.8% and 80.2% of the respective fresh-sand reference strengths. At every corresponding temperature and treatment time, the measured strengths of the mixtures containing 50% thermally reclaimed matrix were lower than those obtained with 100% thermally reclaimed matrix. Thus, unlike the mechanical-reclaim mixtures, partial replacement with fresh sand did not improve bending strength under the thermal-reclamation conditions tested. This indicates that the benefit of fresh-sand addition depends on the condition of the reclaimed matrix and cannot be generalized across reclamation routes. Differences in grain packing, surface condition, and binder distribution may contribute to this behavior, but their individual effects were not isolated.

The reported standard deviations, generally ranging from 0.01 to 0.09 MPa, describe the variability of the bending-strength measurements within the tested conditions, rather than run-to-run reproducibility of reclamation. For mechanically reclaimed matrices, the maximum strengths obtained with the 50/50 mixtures were closer to the corresponding fresh-sand reference strengths than those obtained with 100% reclaimed matrix. For thermally reclaimed matrices, however, the 50/50 mixtures had lower measured strengths than the corresponding mixtures containing 100% reclaimed matrix at every matched temperature and treatment time.

Quartz undergoes a reversible α–β transformation at approximately 573 °C at atmospheric pressure, accompanied by a change in volume [19]. The nominal reclamation temperatures used here (600–1000 °C) exceed this threshold; the thermal exposure of the grains should nevertheless be assessed using the measured bed-temperature histories in Figure 8. Transformation-related strain, together with thermal gradients and pre-existing grain defects, may promote microcracking or chipping during heating and cooling. Although the crystallographic transformation is reversible, any resulting mechanical damage need not be.

Possible changes in grain angularity, surface condition, and fines content could modify packing, binder demand, and binder-bridge formation, thereby influencing the bending strength of molding sands prepared with thermally reclaimed matrix. These effects may coexist with the beneficial removal of residual binder. The extent of these effects was not determined in this study because paired grain-size distributions of the matrix, SEM observations before and after thermal treatment, and measurements of thermal dust losses were not available. The proposed mechanisms should therefore be regarded as potential contributions rather than experimentally established explanations of the observed strength differences.

From an industrial perspective, the tested 50/50 blend provides a promising starting point for reuse trials but does not establish the maximum acceptable reclaimed-sand proportion. Mechanical reclamation avoids the high-temperature heating step, although residual binder and fines may limit reuse. For fine binder-jetting sand, classification must balance fines removal against the loss of useful grains, while possible fragmentation may affect binder demand and spreading behavior. Thermal reclamation achieved lower residual LOI, but its practical benefits must be assessed against energy demand, off-gas treatment, and possible grain damage. Consequently, acceptable reclaimed-sand proportions for either route require application-specific validation of mold performance and casting quality, including repeated reclamation cycles.

## 4. Conclusions

This study demonstrated the potential for reclaiming spent furfuryl-resin molding sands produced by binder jetting and conventional molding. The results should be interpreted within each sand system, considering differences in initial material characteristics and the applied air-classification conditions. The main conclusions are as follows:

− Mechanical reclamation reduced LOI within both sand systems as rotor speed and treatment time increased. At 560 rpm for 30 min, LOI decreased from 2.70% to 1.85% for US-1 and from 2.85% to 1.68% for US-2, corresponding to WRK values of 31.5% and 41.1%, respectively. These results characterize the tested material–process combinations and do not establish the superiority of either mold-manufacturing technology.
− The collected dust fraction increased with the intensity of mechanical treatment. Different classification velocities—0.75 m/s for US-1 and 1.0 m/s for US-2—limit direct comparisons of dust content, retained-matrix LOI, and WRK. Dust measurements alone do not distinguish newly fractured grains from pre-existing fines released during cleaning.
− Within each sand system, thermal reclamation achieved lower residual LOI than mechanical treatment, reaching 0.14% for US-1 and 0.29% for US-2 at the highest nominal temperature after 30 min. For mixtures containing 100% reclaimed matrix, the maximum bending strengths after thermal reclamation were 4.43 and 3.34 MPa, respectively, compared with 3.69 and 2.58 MPa after mechanical reclamation. These improvements do not exclude simultaneous thermal damage to the grains.
− Mixtures containing 50% mechanically reclaimed matrix and 50% fresh sand achieved maximum bending strengths of 4.69 MPa for US-1 and 3.45 MPa for US-2. These values represent 83.6% and 88.7% of their respective fresh-sand reference strengths of 5.61 and 3.89 MPa. US-1 therefore achieved the higher absolute maximum strength, whereas US-2 retained a larger fraction of its own reference strength.
− Fresh-sand addition did not provide the same benefit for thermally reclaimed matrices. The mixtures containing 50% thermally reclaimed matrix and 50% fresh sand reached maximum strengths of 4.14 MPa for US-1 and 3.12 MPa for US-2, corresponding to 73.8% and 80.2% of their respective fresh-sand reference strengths. At every corresponding temperature and treatment time, their measured strengths were lower than those of mixtures containing 100% thermally reclaimed matrix. The benefit of partially replacing reclaimed matrix with fresh sand therefore depends on the reclamation route and matrix condition; a universal optimum at a 50/50 ratio cannot be inferred.
− Practical process selection requires consideration of losses of useful grains, grain integrity, energy demand, and off-gas treatment alongside bending strength and residual LOI. Application-specific reuse limits require further assessment of mold performance and casting quality, additional blend ratios, and repeated reclamation cycles.

## Figures and Tables

**Table 1:** Composition of the molding mixtures used to prepare the BJ and conventional molds.

Sand System	Resin, %	Hardener, %	Hardener/Resin
Additive BJ	2.50	0.75	0.30
Conventional mold production	2.00	1.00	0.50

**Table 2:** Properties of the spent sands before secondary reclamation. LOI—loss on ignition; dg—geometric mean grain diameter; da—arithmetic mean grain diameter; Mf—main fraction content; main fraction—the sizes of the adjacent sieves in the standard sieve set that together retained the largest proportion of the tested material.

Spent Sand	LOI [%]	pH	dg [mm]	da [mm]	Mf [%]	Main Fraction [mm]
US-1	2.70	3.96	0.125	0.141	90.6	0.100/0.071/0.160
US-2	2.85	3.67	0.287	0.332	80.6	0.200/0.160/0.320

**Table 3:** Mean grain diameters of sands reclaimed under different mechanical-reclamation conditions, measured after final classification.

Mechanical-Reclamation Conditions	Reclamation Time [min]	US-1: d_a_ [mm]	US-1: d_g_ [mm]	US-2: d_a_ [mm]	US-2: d_g_ [mm]
Fresh sand	—	0.1378	0.1154	0.3172	0.2808
Before reclamation (reference)	—	0.1419	0.1259	0.3323	0.2877
280 rpm	10	0.1409	0.1227	0.3273	0.2852
280 rpm	20	0.1395	0.1192	0.3229	0.2835
280 rpm	30	0.1386	0.1175	0.3189	0.2812
420 rpm	10	0.1393	0.1209	0.3245	0.2837
420 rpm	20	0.1372	0.1165	0.3185	0.2811
420 rpm	30	0.1342	0.1137	0.3125	0.2765
560 rpm	10	0.1384	0.1182	0.3212	0.2824
560 rpm	20	0.1338	0.1132	0.3138	0.2776
560 rpm	30	0.1266	0.1096	0.3043	0.2643

**Table 4:** Bending strength of molding sands prepared with fresh silica sand and mechanically reclaimed matrices.

Reclamation Time [min]	Fresh Silica Sand [MPa]	280 rpm [MPa]	420 rpm [MPa]	560 rpm [MPa]
US-1—100% mechanically reclaimed matrix				
10	5.61 ± 0.05	1.13 ± 0.02	1.32 ± 0.03	1.95 ± 0.06
20	5.61 ± 0.05	1.50 ± 0.03	1.67 ± 0.04	2.75 ± 0.07
30	5.61 ± 0.05	1.65 ± 0.04	2.11 ± 0.06	3.69 ± 0.08
US-2—100% mechanically reclaimed matrix				
10	3.89 ± 0.03	0.89 ± 0.01	0.93 ± 0.06	1.91 ± 0.05
20	3.89 ± 0.03	1.19 ± 0.02	1.69 ± 0.03	2.23 ± 0.04
30	3.89 ± 0.03	1.55 ± 0.03	2.17 ± 0.09	2.58 ± 0.06
US-1—50% mechanically reclaimed matrix				
10	5.61 ± 0.05	1.63 ± 0.01	1.82 ± 0.03	2.65 ± 0.01
20	5.61 ± 0.05	1.80 ± 0.08	1.97 ± 0.01	3.75 ± 0.04
30	5.61 ± 0.05	1.95 ± 0.05	2.68 ± 0.05	4.69 ± 0.02
US-2—50% mechanically reclaimed matrix				
10	3.89 ± 0.03	1.99 ± 0.02	2.89 ± 0.03	1.89 ± 0.02
20	3.89 ± 0.03	2.34 ± 0.01	3.26 ± 0.01	2.62 ± 0.04
30	3.89 ± 0.03	2.98 ± 0.03	3.45 ± 0.04	3.05 ± 0.03

**Table 5:** Bending strength of molding sands prepared with fresh silica sand and thermally reclaimed matrices.

Reclamation Time [min]	Fresh Silica Sand [MPa]	600 °C [MPa]	800 °C [MPa]	1000 °C [MPa]
US-1—100% thermally reclaimed matrix				
10	5.61 ± 0.05	2.13 ± 0.02	2.32 ± 0.03	2.95 ± 0.06
20	5.61 ± 0.05	2.50 ± 0.03	2.67 ± 0.04	3.75 ± 0.07
30	5.61 ± 0.05	2.95 ± 0.04	3.11 ± 0.06	4.43 ± 0.08
US-2—100% thermally reclaimed matrix				
10	3.89 ± 0.03	1.89 ± 0.01	1.93 ± 0.06	2.22 ± 0.05
20	3.89 ± 0.03	2.19 ± 0.02	2.69 ± 0.03	2.98 ± 0.04
30	3.89 ± 0.03	2.87 ± 0.03	3.17 ± 0.09	3.34 ± 0.06
US-1—50% thermally reclaimed matrix				
10	5.61 ± 0.05	1.89 ± 0.04	2.16 ± 0.04	2.55 ± 0.04
20	5.61 ± 0.05	2.12 ± 0.07	2.22 ± 0.05	3.23 ± 0.04
30	5.61 ± 0.05	2.28 ± 0.01	2.92 ± 0.05	4.14 ± 0.04
US-2—50% thermally reclaimed matrix				
10	3.89 ± 0.03	1.56 ± 0.05	1.23 ± 0.05	2.12 ± 0.02
20	3.89 ± 0.03	1.89 ± 0.05	1.74 ± 0.05	2.58 ± 0.01
30	3.89 ± 0.03	2.19 ± 0.03	3.09 ± 0.02	3.12 ± 0.04

## Data Availability

The original contributions presented in this study are included in the article. Further inquiries can be directed to the corresponding author.
